# Widespread terrestrial ecosystem disruption at the onset of the Paleocene–Eocene Thermal Maximum

**DOI:** 10.1073/pnas.2509231122

**Published:** 2026-01-20

**Authors:** Mei Nelissen, Debra A. Willard, Han van Konijnenburg-van Cittert, Gabriel J. Bowen, Teuntje Hollaar, Appy Sluijs, Joost Frieling, Henk Brinkhuis

**Affiliations:** ^a^Department of Ocean Systems, Royal Netherlands Institute for Sea Research, Den Burg 1790 AB, the Netherlands; ^b^Department of Earth Sciences, Laboratory of Palaeobotany and Palynology, Faculty of Geosciences, Utrecht University, Utrecht 3584 CB, the Netherlands; ^c^Florence Bascom Geoscience Center, United States Geological Survey, Reston, VA 20192; ^d^Department of Geology and Geophysics, University of Utah, Salt Lake City, UT 84112; ^e^Department of Earth Sciences, University of Oxford, Oxford 3584 CB, United Kingdom; ^f^Department of Geology, Ghent University, Ghent 9000, Belgium

**Keywords:** carbon cycle, ferns, terrestrial feedback mechanism, climate change, Paleocene–Eocene Thermal Maximum

## Abstract

The Paleocene–Eocene Thermal Maximum (PETM, ~56 Mya) interval was marked by massive ^13^C-depleted carbon emissions into the ocean/atmosphere system, manifested as a negative carbon isotope excursion (CIE) in sedimentary components, and ~5 °C global average warming. Episodes of hydrological perturbations and soil-erosion have been widely documented for the PETM but their link with vegetation- and carbon cycle changes remain poorly constrained. Here, we present organic microfossil evidence showing a strong increase in fern-dominated pioneer vegetation that replaced coniferous forests on the margin of the Norwegian Sea during the first millennia of the CIE. With the present stratigraphic constraints, the “fern spike” occurred simultaneously in terrestrial settings along the North Sea, Arctic Ocean, the US east coast and in southern Australia, indicating that pioneer vegetation persisted for several millennia following a partial collapse of previously stable terrestrial ecosystems. Both the ferns and influx of microcharcoal imply recurrent physical disturbance, including soil destabilization and erosion, potentially linked to droughts, wildfires, and strong hydrological forcing resulting from extreme climate change. Together with evidence for reworked clay minerals and ancient organic matter (kerogen), these findings show that highly disturbed terrestrial ecosystems were widespread across mid- and high-latitude regions globally. Carbon cycle model simulations suggest that a substantial loss of standing and buried biomass, along with oxidation of soil organic matter, acted as important positive feedbacks during the onset of the CIE. Additionally, enhanced kerogen weathering likely contributed as another major positive feedback throughout both the onset and main phase of the CIE.

## Significance

The Paleocene–Eocene Thermal Maximum (PETM; ~56 Ma) was marked by rapid global warming, making it a valuable test bed for the effects of extreme climate change on the environment. Using pollen and spores preserved in a laminated sedimentary sequence, we reconstruct vegetation change at decadal time-scales. Our results, integrated with existing vegetation reconstructions, reveal a widespread geologically synchronous shift to highly disturbed terrestrial ecosystems and biomass loss, that occurred within decades to centuries after massive carbon release during the PETM-onset and lasted millennia. Modeling suggests that carbon release from such perturbed terrestrial reservoirs, including biomass, soils, and buried kerogen, acted as significant positive feedback, underscoring the need to include land carbon reservoirs in future (PETM) carbon cycle assessments.

The Paleocene–Eocene Thermal Maximum interval (PETM; ~56 Mya) is marked by a distinct negative carbon isotope excursion (CIE) signaling the massive input of ^13^C-depleted carbon into the ocean-atmosphere system (1). The onset of the PETM is characterized by a 3 to 4‰ decrease in global exogenic δ^13^C that likely took place in only 1 to 5 ky (2, 3). The relatively rapid onset of the CIE is followed by a 100 to 170 ky “body” phase of quasistable low δ^13^C values and a 50 to 135 ky long recovery (4–6). Globally, the PETM is characterized by climate warming (7–9), associated with major hydrological perturbations (10, and references therein), episodic/seasonal precipitation extremes (e.g., refs. 10–12), vegetational changes (13), changes in wildfire regime (14), and increases in eroded sediment supply to marginal seas (15).

During the PETM, climate-related disturbances to terrestrial carbon reservoirs may have operated as net positive carbon cycle feedback mechanisms, for example, through the widespread loss of biomass and erosion of (organic-rich) soils and sedimentary rocks (16–18). Although vegetation- and soil-associated carbon reservoirs have even been invoked as a cause of (or contributing to) the CIE (19) as well as aspects of its recovery (20), the precise timing and magnitude of terrestrial disturbances and their importance on the global scale remain elusive. Crucially, the relation of terrestrial carbon cycle feedback mechanisms such as biomass and soil loss to the short-lived CIE-onset phase has so far not been assessed. Stratigraphically expanded sediment sections are a prerequisite for such detailed CIE-onset studies, but these records are sparse. Depositional processes such as bioturbation and other sediment mixing processes may further complicate the identification of the exact position of the CIE onset (3).

Here, we utilize a unique, stratigraphically expanded, annually microlaminated CIE onset-interval recovered from the Norwegian Margin (21) to study the vegetation response during the CIE onset at a decadal-centennial resolution. We complement this study with a dataset recently generated on similarly expanded though nonlaminated sections from the US Atlantic Coastal Plain (22). By employing marine and terrestrial organic walled microfossil assemblages, we reconstruct vegetational changes and (micro)charcoal abundances to assess the role of wildfires. To evaluate terrestrial disturbances during the CIE onset globally, we align various (published) records (22–27) and explore their potential importance for the global carbon cycle with a carbon cycle model (16).

## Results

**Fig. 1.**
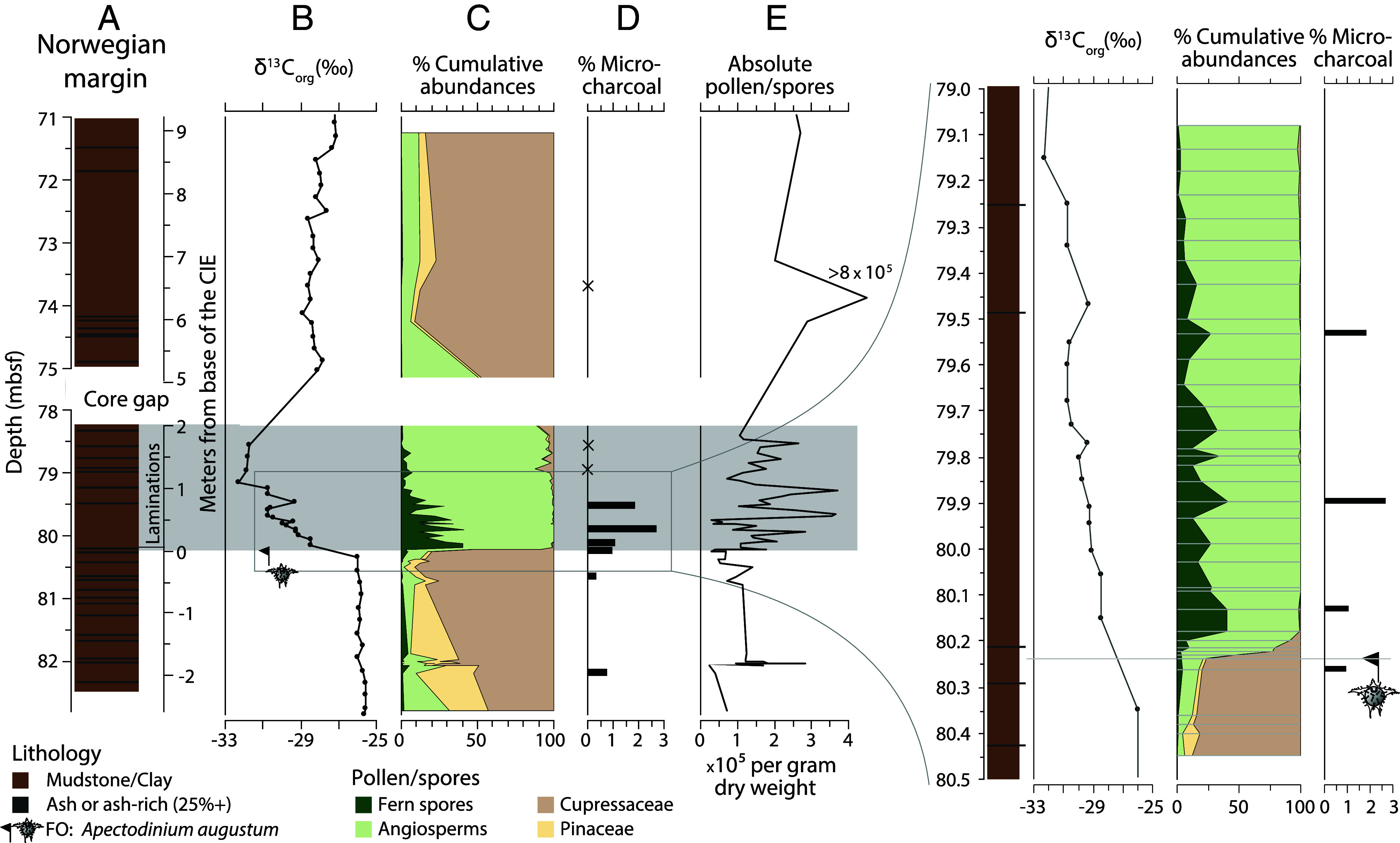
CIE onset and fern spike in Hole U1567B (Norwegian margin). (*A*) Lithology showing ash layers and laminated interval indicated by light gray bar. (*B*) Based on the FO of *A. augustum* [updated from Berndt et al. (32) and bulk δ^13^C_org_ (31)], the depth of the base of the CIE is placed at 80.24 mbsf. (*C*) Cumulative abundances of ferns spores, angiosperms, Cupressaceae, and Pinaceae represent their abundance as a percentage of the total pollen and spores assemblage. (*D*) Microcharcoal abundances given as % of total palynofacies, “x” indicates samples where microcharcoal was absent. (*E*) Absolute concentrations of pollen and spores are given per gram dry weight.

The first occurrence (FO) of the PETM dinocyst species *Apectodinium augustum* at 80.240 ± 0.005 m below sea floor (mbsf) coincides with the onset of the laminated interval, represented by a sample 53.5 to 54.5 cm from the top of core 11X section 2 in Hole U1567B, drilled during International Ocean Discovery Program (IODP) Expedition 396 at the Norwegian Margin (Fig. 1) (21, 28–31). This depth coincides precisely with the first recorded drop in total organic carbon δ^13^C, which represents the base of the CIE. We distinguish this base from the “CIE onset,” which is the several millennia long phase in our record with decreasing δ^13^C values. Below the base of the CIE in U1567B, pollen derived from coniferous vegetation (notably members of the family Cupressaceae, such as *Taxodium* and *Metasequoia,* and the Pinaceae, such as *Pinus* and *Picea*) dominate the assemblage (Fig. 1). Within a 5 (min)–7.5 (max) cm interval above the base of the CIE (80.240 ± 0.005 m to 80.1775 ± 0.0075 m, including a 1.5-cm-thick tephra), a sharp increase in fern spores (hereafter “fern spike”) to ~40% of the terrestrial palynomorph assemblage is observed, along with an increase in angiosperms (see *SI Appendix* for photo plates and full discussion). Absolute fern spore counts show a similar signal, increasing by 70-fold from <10^3^ g^−1^ dry sediment in the pre-CIE onset assemblage to >5 × 10^4^ g^−1^ during the CIE onset (*SI Appendix*, Fig. S1), while the total pollen and spore count shows little change (1 to 3 × 10^5^ g^−1^ see Fig. 1 and *SI Appendix*, Fig. S1). In stark contrast to the ferns, Cupressaceae and Pinaceae pollen counts decline from >10^5^ g^-1^ in the pre-CIE assemblage to <200 g^−1^ during the CIE onset. We interpret these signals to predominantly represent actual changes in vegetation, rather than changes in taphonomy (see *SI Appendix* for full discussion).

The vast majority (70 to 80%) of the fern spores represent the ground/soil-covering, rhizome-forming Gleicheniaceae, which are characteristic for open, disturbed habitats (33, 34) and fire-prone (paleo) environments (35–37). Within these same samples, we find a corresponding increase in the relative abundance of microcharcoal particles to 2.6% of the palynofacies, which is subsequently absent in the body of the CIE (Fig. 1). The close affinity of Gleicheniaceae and wildfires and the presence of microcharcoal implies enhanced wildfire activity directly following the CIE onset. Along with the fern spike, the low diversity angiosperm assemblage is dominated by *Alnipollenites* (alder-type wetland plant), *Ulmipollenites* (elm) (see *SI Appendix*, Figs. S2–S5 for photo plates). Living relatives of *Alnipollenites* are nitrogen-fixing early colonists in nutrient-deficient soils (38) that have been recorded in association with post-landslide soils (39). Both alder and elm thrive along river banks and on flood plains (40, 41). Throughout the entire laminated interval, we observed frequent *Platycarya,* a genus successful at colonizing open or unstable ground (32). Following the fern spike, in the upper part of the laminated interval, assemblages are dominated by angiosperms (including *Caryapollenites* and other juglandaceous trees), representing a broad-leaved forest. Based on the ecological preferences of living relatives of *Caryapollenites,* this vegetational shift is consistent with the establishment of better-drained floodplains. The coniferous mature forest vegetation, representing late successional swamp climax vegetation similar to the vegetation that disappeared at the CIE onset, was reestablished during the later stages of the CIE, which indicates a return to more stable conditions.

## Discussion

### Widespread, Geologically Rapid, and Near-Simultaneous Shift to Highly Disturbed Vegetation.

We can use the U1567B record to acquire crucial temporal constraints on the rate of the shift from coniferous to fern-dominated vegetation that is recorded between 80.240 and 80.1775 mbsf (including a 1.5-cm-thick tephra). This shift was associated with a dramatic change in palynofacies, marked by high amounts of leaf cuticles and plant remains, along with very low absolute pollen counts, indicating substantial erosion on terrestrial surfaces (see *SI Appendix*, Fig. S2 for a detailed overview of palynofacies changes within this interval). Although the analyzed succession records rapid changes, several lines of evidence make a hiatus highly unlikely. The sediment sequence is visually continuous, with a well-preserved pre-CIE interval that includes laminations and has been dated to the latest Paleocene by biostratigraphy (31). Frequent mm-scale ash layers occur throughout the latest Paleocene and into the CIE onset, demonstrating undisturbed deposition that would not persist under bioturbation, erosion, or current activity in soft shallow marine sediments, further supporting near-continuous deposition.

Given these 3.5 to 6 cm of sediment and sedimentation rates of 20 to 40 cm ky^−1^ (*Materials and Methods*), the vegetation shift occurred within maximally 300 y but could have also occurred within days to months in response to extreme events following fire and/or weather extremes that cleared vegetation from a large source area. Modern wildfires and floods can impact regions larger than 100,000 km^2^ (42, 43), with fires often persisting for months (44) emphasizing the severity and spatial scale of these subannual events and their impact. Following the vegetation shift, the subsequent ~1 m thick interval is dominated by Gleicheniaceae, *Ulmipollenites*, and *Alnipollenites*, which indicates a heavily disturbed landscape, affected by increased wildfire activity and flooding. These assemblages indicate colonization after recurrent flooding events, as observed in other Eocene analogs (45). Moreover, this open landscape with exposed soils signifies a terrestrial environment susceptible to enhanced soil carbon and biomass losses (46–49). Taken together, we interpret this ~1 m thick interval to reflect a continuously (prolonged and/or frequently) disturbed habitat with open vegetation. Crucially, these changes happened within decades to centuries following the base of the CIE and the fern-dominated vegetation lasted for millennia. Such a rate of change is broadly consistent with vegetation response times of <200 y recorded in response to abrupt climate changes including rapid warming at the termination of the Younger Dryas (50) and late glacial climate oscillations (51).

**Fig. 2.**
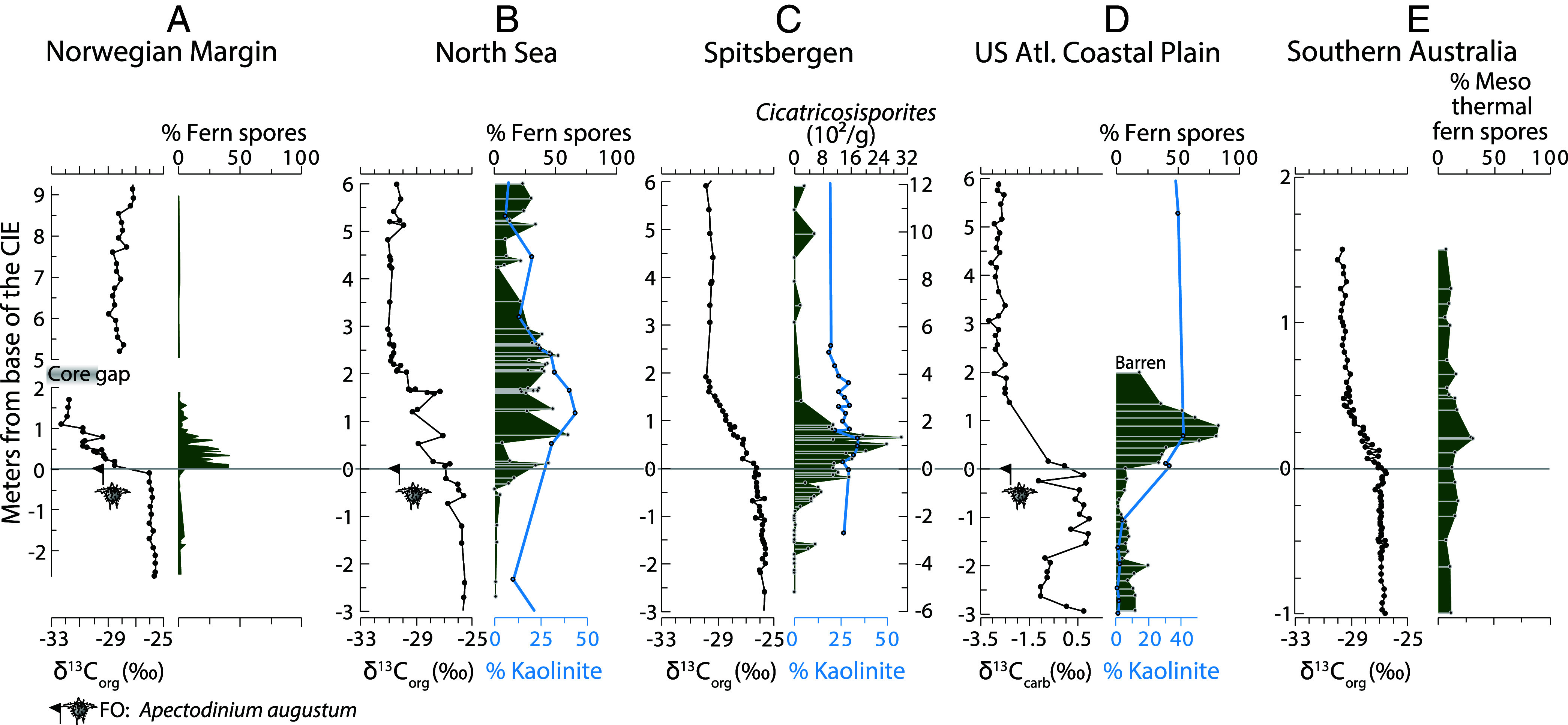
Carbon isotope values (δ^13^C), fern spore abundances (green silhouette), and clay mineralogy [%kaolinite of total clay fraction (blue line)]. Five marginal marine successions with detailed C-isotope and biostratigraphic constraints are shown: (*A*) the Norwegian Margin (Hole U1567B, this study), (*B*) the North Sea [Hole 22/10a, (23, 60)], (*C*) Spitsbergen [δ^13^C and fern counts from the Longyearbyen section, depth axis on the left (24) and kaolinite data from BH9/05, right-hand depth axis (26)], (*D*) US Atlantic Coastal Plain [SDB, (22, 61)], and (*E*) southern Australia (Point Margaret, 38). The horizontal line indicates the base of the CIE. Cores have been aligned with the base of the CIE at 0 m, based on the FO of *A. augustum* and δ^13^C decline. See *SI Appendix* for full discussion of tie points.

Crucially, the FO of *A. augustum* and detailed carbon isotope chemostratigraphy enable correlation of the CIE base with other records and place the events in a global context. In addition to the fern spike recorded in Hole U1567B on the Norwegian Margin (this study), a likely time-equivalent interval of abundant fern spores was recently found along the US Atlantic Coastal Plain [(22, 52); and Fig. 2]. Moreover, reassessment of previous work indicates similar high fern abundance in records from the North Sea (23), Spitsbergen (24), and southern Australia (27) (Fig. 2). In these records, the base of the CIE and, where present, the FO of *A. augustum* allow robust stratigraphic correlation between these records. Although the duration of the fern spikes in the other records cannot be constrained to the same detail as in Hole U1567B, the estimated durations are all approximately several millennia based on published sedimentation rate estimates (*Materials and Methods*) (53–56). The highest abundances of ferns for each locality reach a maximum within 1 to 5 ky following the base of the CIE. The most parsimonious explanation is that these fern spikes were geologically synchronous to within at most a few millennia following the start of anomalous ^13^C-depleted carbon input that defines the base of the CIE. Although fern spore abundance peaks during the onset of the CIE, fern spore abundance increases prior to the CIE in the North Sea and Spitsbergen (Fig. 2), which may link to pre-CIE warming and environmental changes such as those recorded in other records globally (57–59).

On Spitsbergen, no pollen and spore data are available other than quantitative counts of *Cicatricosisporites* spp. These schizaeaceous ferns (62, 63), which also are abundant at the US Atlantic Coastal Plain (22), are indicative of an ecosystem adapted to fire disturbance (64). Similarly, in southern Australia, the fire-prone Gleicheniaceae comprise >10% of the total pollen and spores assemblage during the increase in fern spores (27). These mid-latitude records from both hemispheres are in line with our evidence for increased wildfire activity during the CIE onset at the Norwegian Margin. Notably, the North Sea records a similar successional pattern, with a relative increase in *Alnipollenites* during the fern spike followed by an increase in *Caryapollenites* (23) (*SI Appendix*, Fig. S6). In addition to these records, an increase in fern spores in the lower part of the CIE is tentatively identified in other marine and terrestrial records including in the Bighorn Basin [Wyoming, USA; (65), and New Zealand (66)], although limited microfossil- or carbon isotope data hamper high-confidence alignment to the records presented in Fig. 2.

**Fig. 3.**
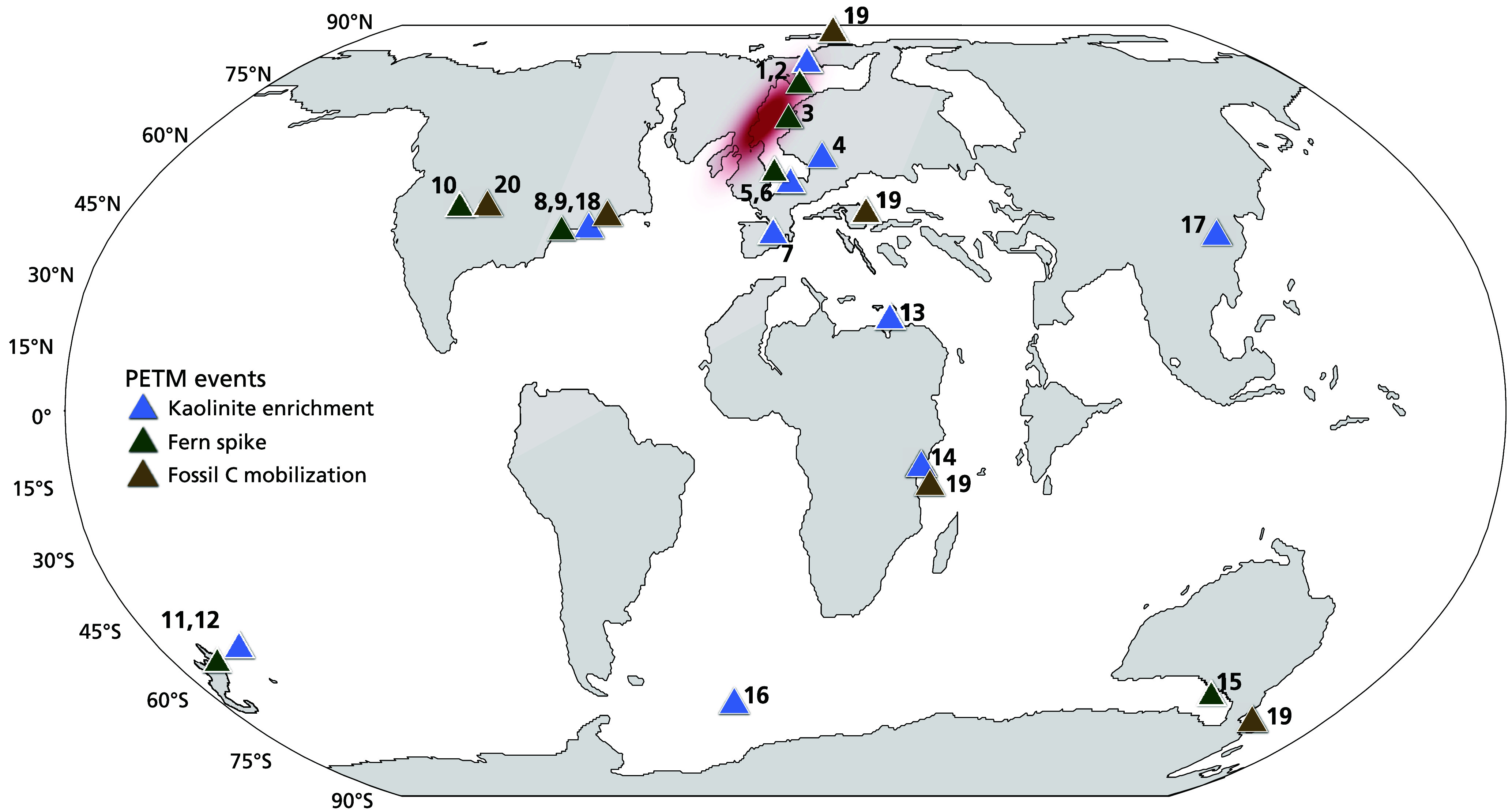
Map of locations where kaolinite enrichment (blue triangle), a fern spike (green triangle), or fossil carbon mobilization (brown triangle) has been recorded during the PETM. The red area indicates the location of the North Atlantic Igneous Province (NAIP). Numbers refer to sites listed in *SI Appendix,* Table S2. Paleogeographic reconstructions at 56 Ma, map adapted from Carmichael et al. (80). Data from references: 17, 18, 23, 24, 26, 27, 60, 61, 65, 66, and 81–86.

Taken together, these records show that the fern spikes following the base of the CIE, within present constraints, were simultaneous and widespread in the mid- and high latitudes in both hemispheres (Fig. 3). Similarly, the decline of cupressaceous and pinaceous conifers near the base of the CIE (*SI Appendix*, Fig. S6) is a widely recognized feature in mid- and high latitudes, which was previously interpreted to reflect the disadvantage conifers have compared to angiosperms to increase growth rates under higher temperature (67). Moreover, we infer that such large-scale replacement of forests by ferns represents a period with significant ecological disruption as a similar trend is widely recorded from key climatic intervals in the geological record associated with biotic upheaval (68, and references therein), e.g., the Cretaceous–Paleogene (69), the Triassic-Jurassic (70), the end Permian (71) mass extinctions and the Carboniferous (72), and likely the modern (73). The link with the CIE onset suggests that this response was tightly linked to the carbon cycle perturbation and indicates that terrestrial vegetation was potentially acting as a positive feedback to rapid climate change during and immediately preceding the CIE onset. For the PETM interval, potential mechanisms driving vegetation disturbances include amplifications of seasonal temperature and precipitation extremes, as previously proposed based on the North Sea pollen data (74) and more intense rainfall events (10), also particularly during the CIE onset (75). In addition to more extreme rainfall events, an increase in wildfire frequency is recorded regionally within the PETM (14, 76–78). These wildfires may have had a long-term (>decadal time-scales) detrimental effect on the vegetation cover and resulted in greatly accelerated soil erosion rates, like in some modern cases (e.g., ref. 79).

### Terrestrial Disturbance and Soil Erosion.

Vegetation turnover and physical disturbances like erosion or wildfires may have impacted the stability of soils through terrestrial feedback mechanisms. An increase in the abundance of the clay mineral kaolinite that has previously been reported at the US Atlantic Coastal Plain (61) and the North Sea (60), in the same interval where the fern spikes are recorded (Fig. 2), indeed seems to imply a link between the increase in fern spores and soil disturbance. Although the palynological data are of much higher temporal resolution and show particularly strong changes at the onset of the PETM, changes in clay mineralogy and notably an increase in the supply of kaolinite are widespread around the globe during the PETM (Figs. 2 and 3) (80, and references therein). Previously, this enrichment has been interpreted to represent exhumation of ancient (pre-PETM) kaolinitic soils (87–89) and intensified weathering (26, 75, 80). Although kaolinite-bearing sedimentary rocks (from laterite or coal-bearing successions) may also contribute to the increase in kaolinite during the PETM, a rock-derived kaolinite flux would be comparatively small compared to that from the more easily mobilized surficial (soil) deposits. Near-surface kaolinite-rich siliciclastic material is formed in tropical humid climates with year-round precipitation (90, 91), but notably also through clay kaolinization during peat formation in temperate climates (37, 92). As both the environments that are conducive to forming appreciable amounts of kaolinite are rich in vegetation and/or soil organic carbon (93), the increase in kaolinite that is recorded during the PETM in mid- and high-latitude regions does not only imply increased soil erosion rates but also shows a likelihood that organic carbon-enriched deposits were exhumed and eroded. Concrete evidence for increased erosion of organic carbon is the input of fossil carbon reworked from ancient organic-carbon bearing sedimentary rocks in the hinterland, often referred to as kerogen weathering, at multiple shelf- (13, 17, 18, 94) and continental (13, 81) records during the PETM body (Fig. 3). The enhanced delivery of fossil carbon indicates enhanced kerogen weathering and, since a significant portion of the original weathered kerogen can be assumed to break down during transport, it contributed to ^13^C-depleted carbon release (17, 95, 96).

Taken together, we find overwhelming evidence for widespread terrestrial vegetation- and soil disturbances, peaking during the first millennia following the base of the CIE. Although the precise duration potentially varied regionally depending on local climatological factors, the stratigraphic correlation, and sedimentation rates support the globally widespread and simultaneous occurrence of fern-dominated vegetation, indicating highly disturbed terrestrial ecosystems. Our observations also show that conditions only became more stable after millennia, allowing for reestablishment of forested vegetation. This is interesting because it suggests that either the intensity or frequency of extreme weather events decreased after this period, or ecosystems became more resilient to these disturbances, resulting in climax vegetation. Importantly, enhanced erosion and reworking of fossil carbon persisted through much of the PETM (17, 18), indicating ongoing physical disturbance without large-scale vegetation turnover, consistent with previous evidence for unstable land surfaces but limited floral change during the CIE body (65). Summarizing, we find robust empirical evidence for a common sequence of terrestrial ecosystem changes recorded at many marine margins: 1) the rapid collapse of coniferous forests and increase of fern-dominated vegetation before and during the CIE onset 2) followed by at least several millennia of highly disturbed terrestrial ecosystems, enhanced soil kerogen mobilization and oxidation and 3) a gradual shift toward more stable conditions that supported climax vegetation and still allowed elevated rates of kerogen weathering during the body of the PETM.

### Implications for PETM Carbon Cycling.

**Fig. 4.**
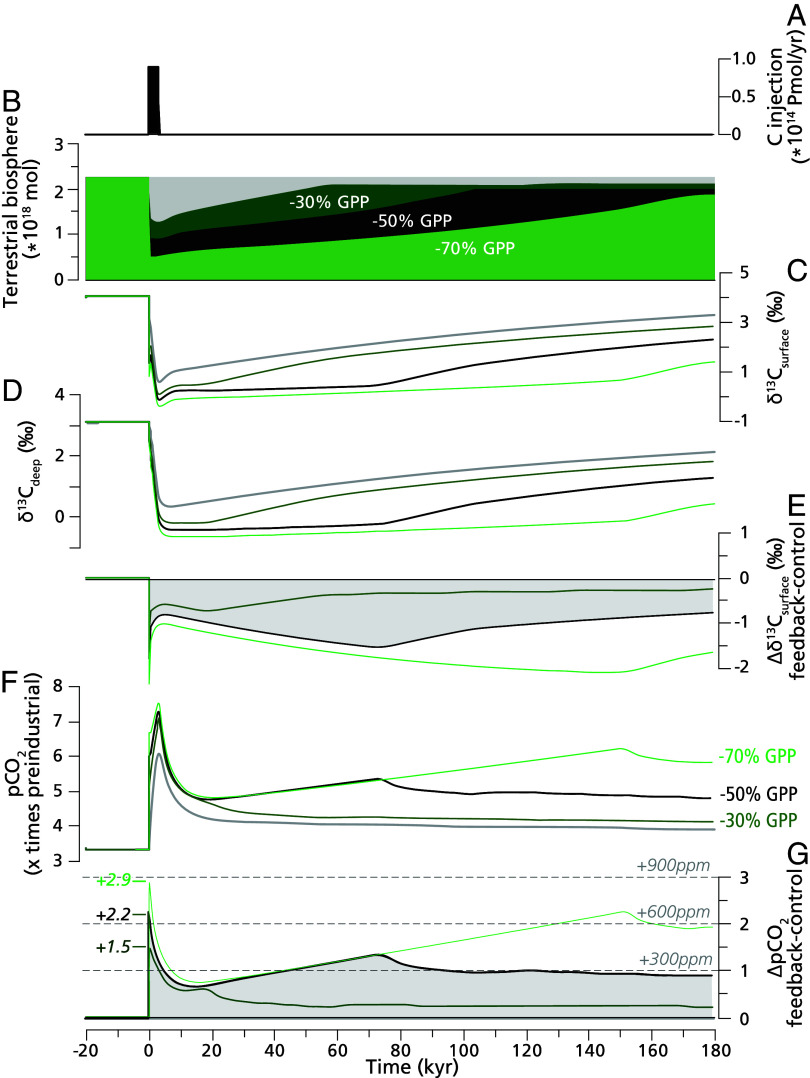
Sensitivity tests of different terrestrial GPP reduction scenarios. Model output data showing. (*A*) the endogenic C that is injected (0.8Pmol y^−1^, corresponding to 0.98Pg y^−1^), (*B*) Three scenarios showing the terrestrial biosphere stock size that result from a reduction in terrestrial GPP of 30% (in dark green, weakest response), 50% (in black), and 70% (in light green, strongest response) in response to endogenic C emissions. The simulated carbon isotope excursions represent (*C*) the global surface, (*D*) deep-water masses, and (*E*) the difference in δ^13^C_surf_ between the control- and feedback scenarios. (*F*) The modeled atmospheric pCO_2_ concentration plotted as x times preindustrial and (*G*) the difference in pCO_2_ concentration plotted as x times preindustrial between the control- and feedback scenarios. The decline in atmospheric pCO_2_ following the spike reflects carbon repartitioning among reservoirs, driven primarily by rapid ocean uptake. The control scenario is indicated with the gray line. The recovery rate for terrestrial GPP for the scenarios shown in this figure was 0.3% per millennium. In *SI Appendix*, we also explore scenarios with faster (1%) and slower (0.1%) GPP recovery rates (*SI Appendix*, Fig. S7). Model details and further discussion are provided in *SI Appendix*.

To assess the potential impacts of the reconstructed terrestrial feedback mechanisms on global carbon cycling, we perform simulations using an adapted version of a box model previously designed to study such feedbacks across the PETM (16) (Fig. 4, model details provided in *SI Appendix*). The largest effects of these feedbacks occur in simulations involving forest collapse during the CIE onset, gradual recovery of terrestrial gross primary productivity (GPP), and increased kerogen oxidation throughout the onset and body of the CIE. In response to the rapid injection of endogenic carbon and consequent disturbance at the CIE onset, we simulate a 50% decrease in terrestrial GPP. This leads to a ~50% reduction in plant and soil carbon storage (Fig. 4). Such reduction in terrestrial carbon storage would transfer ~1,400 PgC from organic stocks to the atmosphere, which amplifies the increase in pCO_2_ by ~600 ppm and the magnitude of the CIE by 1 to 2‰ (Fig. 4). The timescale of biosphere and pCO_2_ recovery is prolonged by the warming-induced increase in respiration rates and kerogen weathering.

These responses are idealized, reflecting the expectation that abrupt global change leads to discrete reductions in ecosystem function, with the severity of impacts scaling with the rate of change (97, 98). The lower GPP bound applied here (50%) is based on observations of reduced organic matter in PETM soils (76, 99), as well as fossil evidence indicating that terrestrial vegetation persisted at PETM sites worldwide. In addition to the 50% decrease in terrestrial GPP, we also tested a more modest terrestrial response with a GPP reduction of 30% (Fig. 4). In this scenario, the increase in CO_2_ is amplified by ~450 ppm, showing that also a weaker response of GPP to endogenic carbon input with only a ~30% reduction terrestrial biomass significantly amplifies the PETM carbon cycle perturbation. In the most extreme scenario, with a GPP reduction of 70%, the increase in CO_2_ is amplified by ~900 ppm. Increased rates of kerogen erosion and weathering, and associated organic carbon respiration feedback, then help sustain elevated atmospheric pCO_2_ (up to ~300 ppm above the simulation without feedbacks) and suppressed δ^13^C values (as much as 1.5‰ lower) throughout the CIE body. We imposed a kerogen weathering feedback in response to changes in the terrestrial biosphere, to reflect the diminished role of vegetation in stabilizing landscapes. This mechanistic link affects the duration of the increase in pCO_2_ during the CIE body (Fig. 4), which stabilizes (and starts to recover) when the terrestrial biosphere has recovered to ~70% of the pre-event value (see *SI Appendix*, Fig. S9 for full discussion). As a result, under the strongest scenario with a 70% reduction in GPP, the kerogen weathering continues to increase pCO_2_ for ~150 ky after the CIE onset. This kerogen weathering feedback in response to changes in the terrestrial biosphere we imposed is highly idealized but produces fluxes that are qualitatively consistent with observations implying increased kerogen weathering during the PETM (17) (see *SI Appendix*, Fig. S8 for all fluxes).

Although the modeled feedbacks are highly parameterized, they serve to illustrate the potential impact of terrestrial carbon stocks in amplifying PETM climate change via changes in state variables that are consistent with the available observational evidence (Figs. 1–3). The results suggest that these processes amplified the severity and duration of PETM global change through the sustained transfer of carbon from terrestrial stocks to the ocean/atmosphere system. Although the responses modeled here are first-order-consistent with observational constraints, several important uncertainties regarding the feedback mechanisms and patterns remain. For example, rates of vegetation recovery following disturbance at the PETM onset are not well constrained (see *SI Appendix*, Figs. S7 and S8 for the model sensitivity to different recovery rates). Also, the degree to which climate change and land cover change each contributed to controlling kerogen weathering rates is not known. These details have substantial effects on the reconstructed persistence, and to some extent the magnitude, of terrestrial carbon cycle feedbacks and warrant further study to better constrain the contribution of terrestrial feedbacks to the PETM carbon cycle and the CIE.

## Conclusions

Pollen and spore data from sedimentary sequences from multiple mid- and high latitude continental margins reveal widespread vegetation- and soil loss during the first millennia of the PETM, a transient global warming phase ~56 Mya with analogies to current climate change. Terrestrial ecosystems partially collapsed within, at most, a few centuries of the onset of the event, likely driven by a combination of wildfires and hydrological climate extremes. Carbon-cycle box-model simulations indicate that carbon released through biomass loss and kerogen oxidation amplified the overall PETM carbon input, particularly during the onset of the event. This amplification persisted beyond the initial phase, as kerogen weathering remained elevated despite the subsequent reestablishment of vegetation. Our data provide a first glimpse into centennial-millennial-scale changes in terrestrial carbon storage in response to (hydro)climate change during a past perturbation of climate and carbon cycle that is relevant to understanding current and future global change. These results imply that vegetation and soils may play a central role in carbon cycle and climate change over the next centuries.

## Materials and Methods

IODP Expedition 396 recovered PETM sediments mainly comprising diatomaceous mudstone from an infilled hydrothermal vent crater located in the North Atlantic Igneous Province (NAIP) on the Norwegian Margin (31, 100). Between 80.24 m and 78.2 m in Hole U1567B (65°21.768′N, 3°3.208′E), the CIE is recorded in bulk organic-matter (δ^13^C_org_) in ~1 m of microlaminated mudstone that includes a number of thin (mm-cm scale) discrete volcanic ash layers, together with the FO of the PETM dinoflagellate cyst marker species *A. augustum* (Fig. 1) (28–30). All data used in this study are provided in *SI Appendix*, Tables S1 and S2 and on Zenodo (10.5281/zenodo.17735745)

### Bulk Organic Matter Carbon Isotope Stratigraphy and Relative Age Constraints.

Organic-matter mixing affects δ^13^C_org_ as Paleogene organic-matter of marine and terrestrial origin had different isotopic compositions (101). This implies that we cannot use the combination of the isotope excursion recorded in δ^13^C_org_ and microlaminated sediments to directly constrain the duration of the onset of the global CIE based on stratigraphic thickness and lamina. However, the lamina themselves allow for independent relative ages for the U1567B succession that records the decline in δ^13^C_org_ starting at the base of the global CIE. The microlaminations are ~100-µm thick and consist of distinct diatom assemblages which were interpreted to represent individual seasonal diatom blooms (21). The laminations are characterized by 2 alternating types of monospecific layers (“blooms”), dominated by either **Hemiaulus* curvatulus* and **Hemiaulus* pungens* or *Grunoviella gemmata* and *Grunoviella* sp. and a layer with mixed diatom assemblages, including large pelagic discoid diatoms (21). This implies 1 seasonal bloom + mixed layer per year (corresponding to 200 µm per year, 20 cm ky^−1^) or 2 seasonal blooms per year + 2 mixed layers, if we interpret the 2 different monospecific layers to each represent 1 seasonal bloom during, e.g., spring and autumn (corresponding to 400 µm per year, 40 cm ky^−1^). Our average sampling interval for the palynological samples is 4 cm with a sample thickness of 0.5 cm, which implies steps of ≤100 y with ≤12.5 y sampled in each 0.5 cm slice or steps of ≤200 y between samples, with ≤25 y sampled in each 0.5 cm slice, assuming a sedimentation rate of 40 cm ky^−1^ or 20 cm ky^−1^, respectively.

### Age-Depth Constraints for Other Successions—Duration of Spikes.

The depth of the base of the CIE, defined by the first anomalously low δ^13^C data point and, where present, the FO of *A. augustum,* were used the align the records from the Norwegian Margin (80.24 mbsf), North Sea (23) (2,613.96 m), Spitsbergen (25) (3.1 m), South Dover Bridge (22) (204.05 m) and Southern Australia (56). Previously published sedimentation rates (24, 53, 55, 56), based on the thickness of the CIE, were used to estimate the duration of the fern spikes. For the North Sea record, we estimated the sedimentation rate based on a thickness of the CIE onset of 2.89 m and an onset duration of 5 ky (3). The calculated duration of the fern spikes following the base of the CIE all range between ~0.7 to 2.1 ky (Southern Australia) and ~5 ky (Spitsbergen). See *SI Appendix*, Table S2 for full discussion per site.

### Organic Walled Microfossil Analyses.

We processed all U1567B samples (n = 73) at the Geolab at Utrecht University following the method described in Sluijs et al. (102). Samples were freeze-dried, gently crushed, and spiked with a known amount of *Lycopodium* spores to quantify microfossil concentrations. The sediment was treated with 30% HCl and 38% HF for carbonate and silica removal, respectively. Residues were sieved using a 10-μm nylon mesh and mounted on slides for palynological analysis. Palynological analyses included counts of pollen and spores derived from terrestrial higher plants and aquatic organic-walled microfossils such as marine dinoflagellate cysts (“dinocysts”). In addition to marine and terrestrial palynomorph analyses, we estimated microcharcoal particle abundances relative to the total palynofacies assemblage for nine of these samples.

### Carbon Cycle Modeling.

We explore the potential carbon cycle impact of the reconstructed vegetation change and other disturbances to the terrestrial carbon cycle using an updated version of the carbon cycle model of Bowen (16). The model represents coupled land–atmosphere–ocean carbon cycle processes and includes feedbacks in terrestrial primary productivity, organic matter respiration, and erosion and weathering of rock kerogen in response to climate destabilization and warming (*SI Appendix*). Model code and scripts used to conduct all model experiments are archived on Zenodo (103) (*SI Appendix*, Software S1).

## Supplementary Material

Appendix 01 (PDF)

## Data Availability

Data have been deposited in Zenodo (https://doi.org/10.5281/zenodo.17735745) (104). All other data are included in the manuscript and/or *SI Appendix*.
