# Supplementary material for: Widespread terrestrial ecosystem disruption at the onset of the Paleocene–Eocene Thermal Maximum
Source: Proc Natl Acad Sci U S A. 2026 Jan 20;123(4):e2509231122. doi: 10.1073/pnas.2509231122 (PMC12849702; doi:10.1073/pnas.2509231122)
Supplement: Supplementary file 1 — Appendix 01 (PDF) [file pnas.2509231122.sapp.pdf]

Supporting Information for

Widespread terrestrial ecosystem disruption at the onset of the Paleocene-Eocene Thermal Maximum

Mei Nelissen*, Debra A. Willard, Han van Konijnenburg-van Cittert, Gabriel J. Bowen, Teuntje Hollaar, Appy Sluijs, Joost Frieling, Henk Brinkhuis

Mei Nelissen
Email: mei.nelissen@nioz.nl

This PDF file includes:

Supporting text
Figures S1 to S9
Table S1 and S2
SI References

Other supporting materials for this manuscript include the following:

Datasets S1
Software S1

Supporting Information Text

Site specific palynological results & discussion

Taphonomy X396

Taphonomy in the broadest sense represents the sum of processes between the production and burial of proxy carriers. This includes (differences in) preservation, sorting, and mixing affecting the fossil record and associated ecological- and biogeographical interpretations (1, and references therein). The bulk of the pollen and spore assemblages in Hole U1567B on the Norwegian Margin were transported to the marine realm by fluvial processes and likely to a lesser extent by aeolian transport. In the studied section, terrestrial palynomorphs are well preserved and abundant with on average ~150,000 grains per gram of dry sediment. The dominance of bisaccate pollen from upland conifers, which produce much more pollen than other taxa and are therefore termed "over-producers", below the base of the carbon isotope excursion (CIE) reflects the presence of conifer-dominated forests in upland sites. In addition, the presence of Cupressaceae pollen in pre-CIE sediments indicates the presence of swamp forests near rivers and/or the coastline (2) in the latest Paleocene (3, 4). If the increase in fern spores following the base of the CIE (Fig. 1, 2) was driven solely by an increase in fluvial transport of spores from land to the depositional site, little change in the absolute numbers of coniferous pollen would be expected. However, concentrations of bisaccate pollen decrease from 5,000-40,000 grains per gram in the pre-CIE assemblages to between 0 and 300 grains per gram during the CIE onset, which can only be explained by either the loss of the coniferous vegetation itself, or cessation of pollen production (Fig. S1) which would quickly result in the loss of coniferous vegetation. The loss of coniferous vegetation is recorded not only at the Norwegian Margin, but at many other records globally based on macrofossils (leaves, cuticles), biomarkers, charcoal mesofossils and palynoflora (5, and references therein). This also supports our hypothesis of a fundamental shift in composition of source vegetation at the onset of the CIE.

Earliest CIE onset and vegetation shift – palynofacies

Plates with microscopic images of the slides shows the clear shift in vegetation and other palynofacies in Hole U1567B core 11X section 2 between 53.5cm and 58.5cm (Fig. S2), the samples directly above the base of the CIE at 80.24 mbsf (11X-2 53.5–54.5 cm). Frequent thin ash layers are visible from the core photos, as well as the onset of the laminated interval at 80.24 mbsf (11X-2 53.5–54.5 cm). The shift in palynofacies supports the vegetation shift and soil disturbance. Very high amounts of leaf cuticles, plant remains and phytoclasts in two samples between the FO of *A. augustum* and the onset of the fern spike, are indicative of substantial erosion and runoff of soils and terrestrial biomass. Comparing these onset samples to the broader shift in palynofacies and palynomorph assemblage from the pre-CIE, CIE onset and CIE body assemblages, shows the remarkable difference with a shift from the Cupressaceae rich assemblages in the pre-CIE and CIE samples, the fern-rich sample with phytoclasts and microcharcoal in the CIE body and the *Caryapollenites* rich interval with high amounts of amorphous organic matter (AOM) in the CIE body from the laminated interval (Fig. S3–S5).

Fern spore abundances at the Cobham Lignite

The five main fern spore abundance records presented in Fig. 2 of the main paper are from the Norwegian Margin, North Sea, Spitsbergen, South Dover Bridge (US east coast) and Point Margaret in southern Australia. An increase in fern spores and an increase in microcharcoal was also recorded from the Cobham Lignite in England in which *Cicatricosisporites* comprised 80% of the total pollen assemblage (6). The stratigraphy of this section however is difficult to link to the other successions presented in Fig. 2. At Cobham, the PETM is identified based on a negative carbon isotope excursion in lignite, which was correlated to an acme of *Apectodinium* in the nearby Woolwich Shell Beds (7, 8). However, the PETM marker dinoflagellate cyst species for the North Atlantic *A. augustum* (9–11) is not present in the Cobham Lignite or the deposits above and below it (6). The presence of the *A. augustum* dinocysts zone (e.g. 12) was proposed based on the *Apectodinium* acme (7) but an *Apectodinium* acme without *A. augustum* does not fit with the characteristics of PETM dinocyst assemblages elsewhere in the North Sea (10, 11, 13–16). Pending further biostratigraphic constraints, it cannot be excluded that the Cobham Lignite represents a younger CIE and *Apectodinium* acme, such as Eocene Thermal Maximum 2 (~54 Ma; (e.g. 17). Considering

the challenging stratigraphy, we do not directly connect the Cobham Lignite to the five main fern-spike records, though we acknowledge that it is possible the Cobham Lignite represents another example of the fern-dominated vegetation that was briefly established during the CIE onset.

A peak in charcoal particles was recorded in the same interval as the highest fern-spore abundance (18). The charcoal remains in the Cobham lignite contained primarily fragments from ferns and flowering plants, which shows the charcoal was derived from the fire- adapted flora itself instead of previously burned pre-PETM vegetation (18). Based on the lack of charred plant reproductive organs, the wildfires were interpreted to have occurred during a distinct season during which plant reproductive organs had not been formed (18). Based on these observations, the authors interpreted these signals to represent episodic fire and runoff, which is in line with our interpretations.

The Fushun Basin records

The Fushun basin records have been previously proposed to record the PETM and subsequent hyperthermals (19–21). However, similar to the age uncertainty of the Cobham lignite, two recent absolute ages based on Ar-Ar (21) and U/Pb (22) for the Lizigou Fm. underlying the Guchengzi Fm. arrive at ages around 55 Ma, suggesting the carbon isotope excursions initially attributed to the PETM may in fact represent later or local events. We tentatively exclude these records from our compilation for this record but emphasize they may further expand the geographical extent of the vegetation changes we observe elsewhere.

Other sites and tie points (Fig. S6)

22/10a, North Sea
In borehole 22/10a (57°44'8.47''N; 1°50'26.59''E), the base of the CIE is placed at 2613.96 m core depth based on the first consistent occurrence (FCO) of *A. augustum,* which is updated from reference (13) who placed the depth of the onset between 2614.3 and 2613.5m based on the $\delta^{13}C$ shift. Samples from a depth of 2619.60 m, 2614.73 m, and 2614.71 m were excluded in the original work as they contained anomalously low $\delta^{13}C$ values and *A. augustum,* representing stratigraphically displaced samples (fall-in) (13). In line with this interpretation, we excluded the fern abundance of three samples (2619.60 m, 2614.73 m, and 2614.71 m) from the data in Fig. 2. Additionally, we have excluded a sample that contained *A. augustum* at a depth of 2617.35m (several meters below the base of the CIE)*,* as *A. augustum* has never been reported from pre-CIE sediments elsewhere and this occurrence is also not supported by any other sample from the same succession. Although this particular sample was not considered a statistical outlier in the original work (13), the current knowledge on the distribution of the PETM marker species *A. augustum* and the displacement of samples from well 22/10a, we find it likely the pre-CIE peak in *A. augustum* represents contamination and not *in situ* specimens. Note that an increase in the genus *Apectodinium* shortly before the CIE onset is recorded at various sites (e.g. 23, 24), but that does not include the species *A. augustum.* Assuming an onset thickness of 2.89 m, based on decreasing $\delta^{13}C_{org}$ values (13) and a maximum onset duration of ~5kyr (c.f. 25). the sedimentation rate for this interval is around 58 cm kyr^{-1}. The thickness of the fern-spike is approximately ~2.77 m, corresponding to a fern-spike duration of around ~ 4.8 kyr.

Spitsbergen
On Spitsbergen, a 14 m thick PETM succession was documented in the Longyearbyen section. We here combined the bulk $\delta^{13}C_{org}$ data and the *Cicatricosisporites* per gram dry weight from this Longyearbyen section (26) with the kaolinite data from the nearby drillcore BH9/05 (27). In BH9/05, the base of the CIE was placed at 533.66 m, corresponding to 3.1 m sampling height in the Longyearbyen section, as previously established (28). Moreover, based on the tie-point between 'bentonite 1' at 10.9 m high in the Longyearbyen section and 517.20 m depth (28), the sedimentation rate in BH9/05 is approximately ~2 times faster during the interval between the base of the CIE and 'bentonite 1' in BH9/05. In the Longyearbyen section, the increase in *Cicatricosisporites* precedes the base of the CIE with ~0.8m and last for ~0.9m into the CIE (26). The observed values of the bulk $\delta^{13}C_{org}$ values are, similar to many other marine marginal settings, affected by mixing of organic matter, resulting in an expanded interval with transitional values at Longyearbyen and BH9/05 (26, 29). Base on a sedimentation rate of BH9/05 ~40 cm kyr^{-1} (30, 31) and assuming 2x slower sedimentation rates in the Longyearbyen section (~20 cm kyr^{-1}), the first increase in

Cicatricosisporites per gram predates the base of the CIE by approximately ~4kyr and the highest absolute counts of *Cicatricosisporites* lasted for ~5kyr following the base of the CIE.

South Dover Bridge, Mayland Shelf
At South Dover Bridge (SDB), Maryland, the base of the CIE was placed at 204.05 m based on the FO of *A. augustum* (32). At SDB, the PETM body and (part of) the recovery span over 15m of sediment. Primary biogenic carbonates are absent and hence no $\delta^{13}C_{carb}$ data is available, which complicates assessment of the base of the CIE. Based on bulk $\delta^{13}C_{org}$ data from the SDB core, decreasing $\delta^{13}C$ values were recorded between 204.25m and 202.39m above which $\delta^{13}C_{org}$ values increase due to an influx of fossil organic carbon (33). Sedimentation on the Atlantic Coastal Plain follows a progradational clinoform depositional model (34), resulting in a more expanded CIE onset in the proximal sites, like SDB. The most proximal site on the Atlantic Coastal Plain, Mattawoman Creek-Billingsley Road (MCBR), experienced sedimentation rates up to 50 cm kyr^{-1} at the CIE onset (35). Self-Trial et al., correlated the first 2.3m of the PETM at MCBR with the basal 1.3m interval at SDB, based on microfossil- and sedimentological data (35). This suggests a sedimentation rate of 28cm kyr^{-1} at SDB for this respective interval. The ~1.2m-thick interval with >30% fern spores would correspond to 4.2kyr.

Point Margaret, Southern Australia
At Point Margaret, the base of the CIE was placed at a depth of 50.8m, based on $\delta^{13}C_{org}$ values and biostratigraphy (*Apectodinium* acme, and first common occurrence of *Florentinia reichartii*) (23). The CIE onset is marked by extensive vegetation turnover recorded between the base of the CIE at 50.8m and 51.3 m. The brief fern-spore peak is recorded in two samples ~20cm above the base of the CIE at 51.0 and 51.05 m and was interpreted to represent a stage of “disturbed vegetation cover”, following the original work (36). Assuming a sedimentation rate of ≥7 cm kyr^{-1} for this interval (23), the stratigraphic interval where the fern-rich vegetation is recorded likely represents a few millennia, in line with the other records we present in this study.

Table S1: overview of calculated durations of fern-spikes and used constraints

Site	Depth or height base of the CIE	Sediment accumulation rate	Fern spike interval thickness	Duration fern spike	References
Norwegian Margin, U1567B	80.24 mbsf $\delta^{13}C_{org,}$ FO *A. augustum*	~20–40 cm kyr-1	~65 cm	~ 1.6 – 3.25 kyr	This study, (37)
North Sea, 22/10a	2613.96 m $\delta^{13}C_{org}$ FO, *A. augustum*	~58 cm kyr-1	~2.77 m	~ 4.8 kyr	This study, (13)
Spitsbergen, Longyearbyen	3.1 m $\delta^{13}C_{org}$	~20 cm kyr-1	~0.8 m (preceding CIE base) ~0.9 m (following CIE base)	~4 kyr (preceding CIE base) ~5 kyr (following CIE base)	(31) , (26), (28)
U.S. Atlantic Coastal Plain, SDB	204.05 m FO *A. augustum*	~28 cm kyr^{-1}	~1.2 m	~ 4.2 kyr	(32, 35)
Southern Australia Point Margaret	50.8 m Apectodinium acme, $\delta^{13}C_{org}$	≥7 cm kyr-1	≥5 cm (max 15 cm)	~0.7 – 2.1 kyr	(23, 36)

Sites presented in Figure 3 (main text)

Table S2: overview of locations, data types and references corresponding to the numbers illustrated in Figure 3 in the main text of this manuscript

Number	Location	Data type	Source
1	Spitsbergen	Cicatricosisporites	Harding et al., 2011 (26)
2	Spitsbergen	Kaolinite	Dypvik et al., 2011 (27)
3	Norwegian Margin	Fern spores	this study
4	Fur, Denmark	Kaolinite	Stokke et al., 2021 (38)

5	North Sea	Fern spores	Kender er al., 2012 (13)
6	North Sea	Kaolinite	Kemp et al., 2016 (39)
7	Zumaya, Spain	Kaolinite	Pujalte et al., 2015 (40)
8	South Dover Bridge, USA	Fern spores	Willard & Brinkhuis, 2025 (41)
9	South Dover Bridge, USA	Kaolinite	Gibson et al., 2000 (42)
10	Bighorn Basin, USA	Fern spores	Korasidis and Wing, 2023 (43)
11	New Zealand	Fern spores	Handley et al., 2011 (44)
12	New Zealand	Kaolinite	Kaiho et al., 1996 (45)
13	Wadi Nukhul, Egypt	Kaolinite	Khozyem et al., 2013 (46)
14	Tanzania	Kaolinite	Handley et al., 2012 (47)
15	Point Margaret, Australia	Fern Spores	Huurdeman et al., 2021 (36)
16	Maud Rise	Kaolinite	Robert and Kennett, 1994; Kelly et al., 2005 (48, 49)
17	Beigou, China	Kaolinite	Chen et al., 2016 (50)
18	South Dover Bridge, USA	Kerogen Weathering	Lyons et al., 2019 (33)
19	ODP Site 1172	Kerogen Weathering	Hollingsworth et al., 2024 (51)
20	Bighorn Basin	Kerogen Weathering	Baczynski et al. 2016 (52)

Supplementary material and methods

Methods C-cycle model

The carbon cycle box model used here was adapted from the work of Walker and Kasting (53) and Archer (54) as previously described (55) and represents the cycling of ^{12}C and ^{13}C in single global deep ocean, surface ocean, atmosphere, and terrestrial biosphere stocks and a 15-layer, depth-differentiated seafloor sediment stack. System inputs include volcanic degassing, weathering of carbonate rocks, kerogen, silicate rocks, and phosphorous, and an imposed injection of ^{13}C-depleted carbon marking the start of the CIE . All weathering fluxes are sensitive to the modeled earth surface temperature, and thus to atmospheric CO_2 concentration, via a climate sensitivity parameter. Outputs include carbon, alkalinity, and phosphorus fluxes associated with marine carbonate burial and organic carbon burial. The former process is a function of the modeled lysocline depth and ocean bathymetry. The latter is a function of organic carbon export from marine and terrestrial systems, determined by marine phosphorous concentration and terrestrial biosphere size, respectively, and a Q_{10} relationship describing the rate of respiration as a function of temperature.

The baseline simulation reported here is identical to the control scenario ("modern" pre-event biosphere stock) modeled by Bowen (55) except that 1) global surface and deep ocean temperature change was modeled as a function of atmospheric CO_2 concentration using a climate sensitivity of 6 °C/doubling (56) rather than prescribed, 2) endogenic carbon release was conducted over a 3,000-year period at a constant rate tuned to achieve a surface ocean CIE of 3.5‰, yielding a total injection of 2,900 Pg C as CO_2, directly into the atmosphere with an assumed $\delta^{13}C$ of -55‰.

The new feedback scenario reported incorporates the temperature-sensitive organic carbon respiration feedback described in Bowen (55) and adds two additional responses to PETM carbon injection. First, we model a decrease in gross terrestrial primary productivity (GPP), in response to carbon injection, with a rate-limited recovery:

$$GPP_t = min\left(GPP_0 \times \left[1 - 0.5\left(\frac{I_t}{I_{max}}\right)^2\right], 1.000003 \times GPP_{t-1}\right), \quad (1)$$

where GPP_t, GPP_{t-1}, and GPP_0 are gross terrestrial primary productivity at model timestep t, $t-1$, and the start of the simulation, respectively, and I_t and I_{max} are the current and maximum endogenic carbon injection

rates. The first term represents a reduction in GPP that scales with injection rate to a maximum of 50% of GPP_0, and the latter limits the rate of recovery of GPP following the CIE onset to 0.3% per millennium. A weaker and stronger response are tested here by also limiting the maximum reduction in GPP to 30% (weaker) and 70% (stronger) of GPP_0. Moreover, we test faster (1% per millennium) and slower (0.1% per millennium) GPP recovery rates (Fig. S7). The modelled fluxes are plotted in Fig. S8, illustrating how terrestrial stock sizes, respiration- and assimilation rates, kerogen weathering and burial fluxes change over time, under the different scenarios that have been tested.

These are highly idealized responses, which reflect the fact that discrete reductions in ecosystem function and changes in terrestrial GPP are expected to accompany abrupt global change and that their severity is thought to scale with the rate of change (57, 58). The minimum bound on GPP used here (50%) is loosely based on observations of the reduced organic matter content of PETM soils (59, 60) and fossil evidence for the persistence of terrestrial vegetation at PETM sites around the globe (i.e., only a partial collapse of productivity). We test the sensitivity of the model results to different lower bounds on GPP_t and recovery rates.

Second, we impose a kerogen weathering feedback in response to changes in the terrestrial biosphere. We parameterize this as threshold effect:

$$kw_t = kw_0\left(2 - \sqrt[3]{Bf_t}\right), \qquad (2)$$

where kw_t and kw_0 are the rate of the organic carbon flux from the lithosphere to the surface ocean (via rivers) at model timestep t and the start of the simulation, respectively, and the biosphere size factor Bf_t is given by:

$$Bf_t = \frac{\left(\frac{B_t}{B_0} - 0.7\right)}{0.3}. \qquad (3)$$

This form allows the weathering flux to tip between two semi-stable states as the size of the biospheric carbon stock decreases below 70% of the initial value (Fig. S9), representing a threshold effect due to loss of landscape-stabilizing vegetation. Again, the response used here is idealized, but produces fluxes that are qualitatively consistent with observations implying increased kerogen weathering during the PETM (33).

Simulations were run for 200 kyr with the first 20 kyr discarded as spin-up. Model code and scripts used to conduct all model experiments are archived on Zenodo (Software S1)

C-cycle model output interpretation and fluxes

As discussed in the main text, the modeled feedbacks are highly parameterized and are intended to illustrate the potential impact of terrestrial carbon stocks in amplifying the CIE. In addition to the sensitivity tests presented in the main text in which we explored weaker and stronger reductions in terrestrial GPP in response to endogenic carbon input, we also tested faster (1% kyr^{-1}) and slower (0.1% per kyr) GPP recovery rates relative to the intermediate recovery rate (0.3% kyr^{-1}) used in the main scenarios. Figure S8 shows the main implications of these differing recovery rates for the duration of the CIE (Fig. S7).

In the fast-recovery scenario, the difference in atmospheric pCO_2 between the control and perturbed runs decreases to nearly zero after ~30 kyr, resulting in a much smaller CIE amplification from the terrestrial feedback mechanism. In contrast, under the slow-recovery scenario, the terrestrial biosphere remains reduced throughout the entire model run, sustaining kerogen weathering and leading to a continued rise in atmospheric pCO_2.

As evident from both the sensitivity experiments (main text Fig. 4; Fig. S7) and the modeled fluxes (Fig. S8), the model output is sensitive to the prescribed mechanistic link between terrestrial biosphere stock size and kerogen weathering (Fig. S9). In all simulations, atmospheric pCO_2 stabilizes once the terrestrial biosphere recovers above ~70% of its pre-CIE size, coinciding with the onset of $\delta^{13}C$ recovery in surface and deep-ocean reservoirs.

Finally, we note that the scenario with a 50% reduction in terrestrial GPP and a recovery rate of 0.3% kyr^{-1} produces a CIE body-to-recovery shape that broadly matches the observed $\delta^{13}C$ pattern, with an ~80 kyr CIE body and >100 kyr recovery (although a full return to pre-CIE values is not achieved in the model). These results highlight the importance of incorporating terrestrial feedback mechanisms in carbon-cycle models.

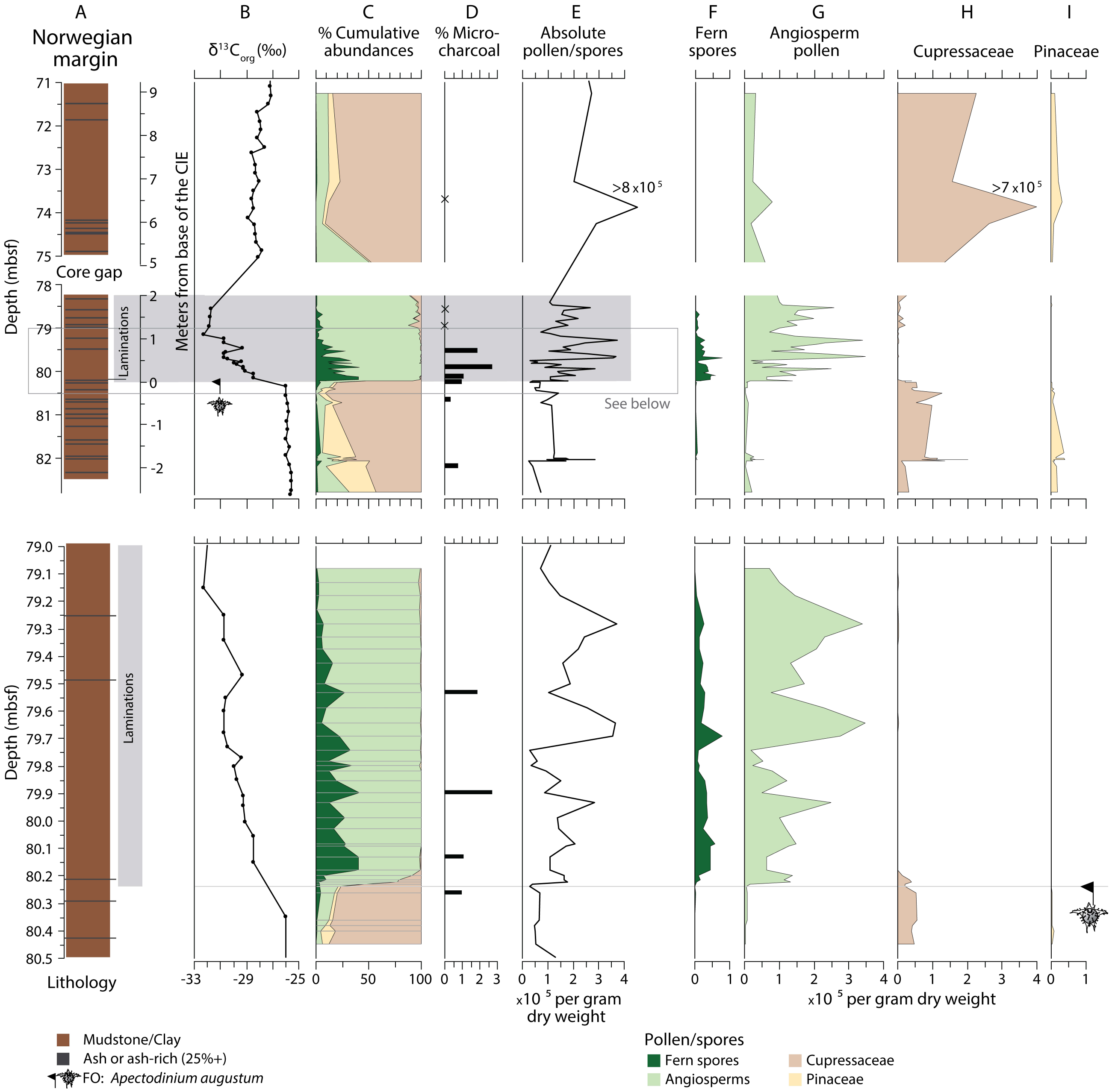

Fig. S1. CIE onset and fern spike in Hole U1567B (Norwegian margin) A) Lithology showing ash layers and laminated interval indicated by light grey bar. B) Based on the FO of A. augustum (updated from Berndt et al., 2023 (37) and bulk $\delta^{13}C_{org}$ (37), the depth of the base of the CIE is placed at 80.24 mbsf. C) Cumulative abundances of ferns spores, angiosperms, Cupressaceae and Pinaceae represent their abundance as a percentage of the total pollen and spores assemblage. D) Microcharcoal abundances given as % of total palynofacies, 'x' indicates samples where microcharcoal was absent. Absolute counts of pollen and spores are given per gram dry weight for E) sum of pollen and spores, F) fern spore counts, G) angiosperm pollen, H) Cupressaceae and I) Pinaceae.

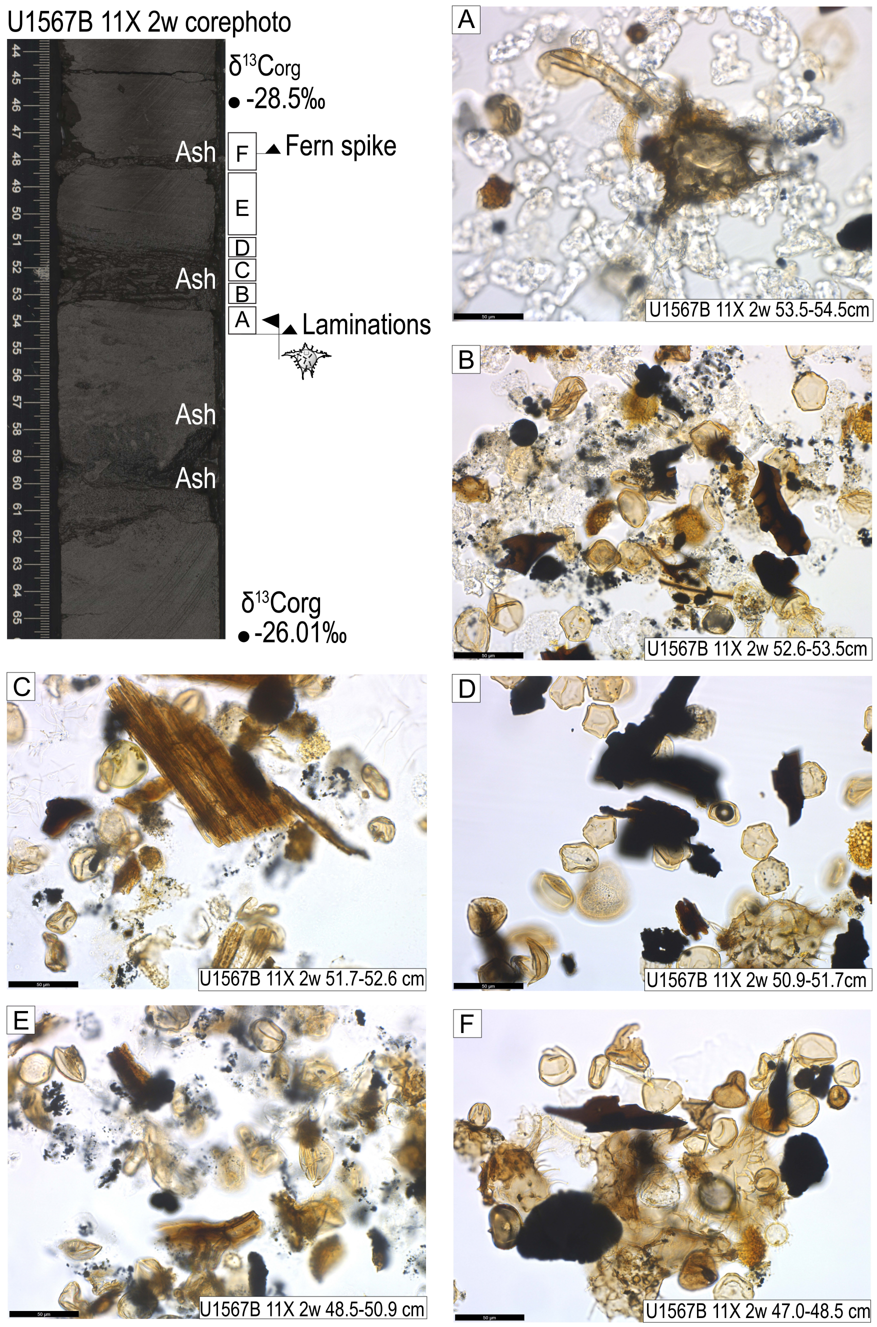

Fig. S2 Corephoto of U1567B and micrographs of palynological preparations from the indicted levels. The corephoto (top left) shows the presence of ash layers and the onset of laminations. Bulk $\delta^{13}C_{org}$ values (37) and FO of A. augustum show the base of the CIE. A) shows the FO of A. augustum, along with remnants of minerals, a potential result of the presence of ash in that sample. B) a mix of minerals, phytoclasts, pyrite and increased amounts of pollen and spores are observed. The pollen and spores are dominated by Cupressaceae pollen. C) high amounts of leaf cuticles, plant remains and phytoclasts, D) high amounts of black phytoclasts and microcharcoal, along with increased amounts of Alnipollenites (red arrow) and Ulmipollenites (blue arrow). E) remnants of minerals, a potential result of the presence of ash in that sample, increased amounts of phytoclasts. F) minerals are absent, and ferns (mostly Gleicheniaceae, red arrow) comprise >40% of the pollen/spores assemblage.

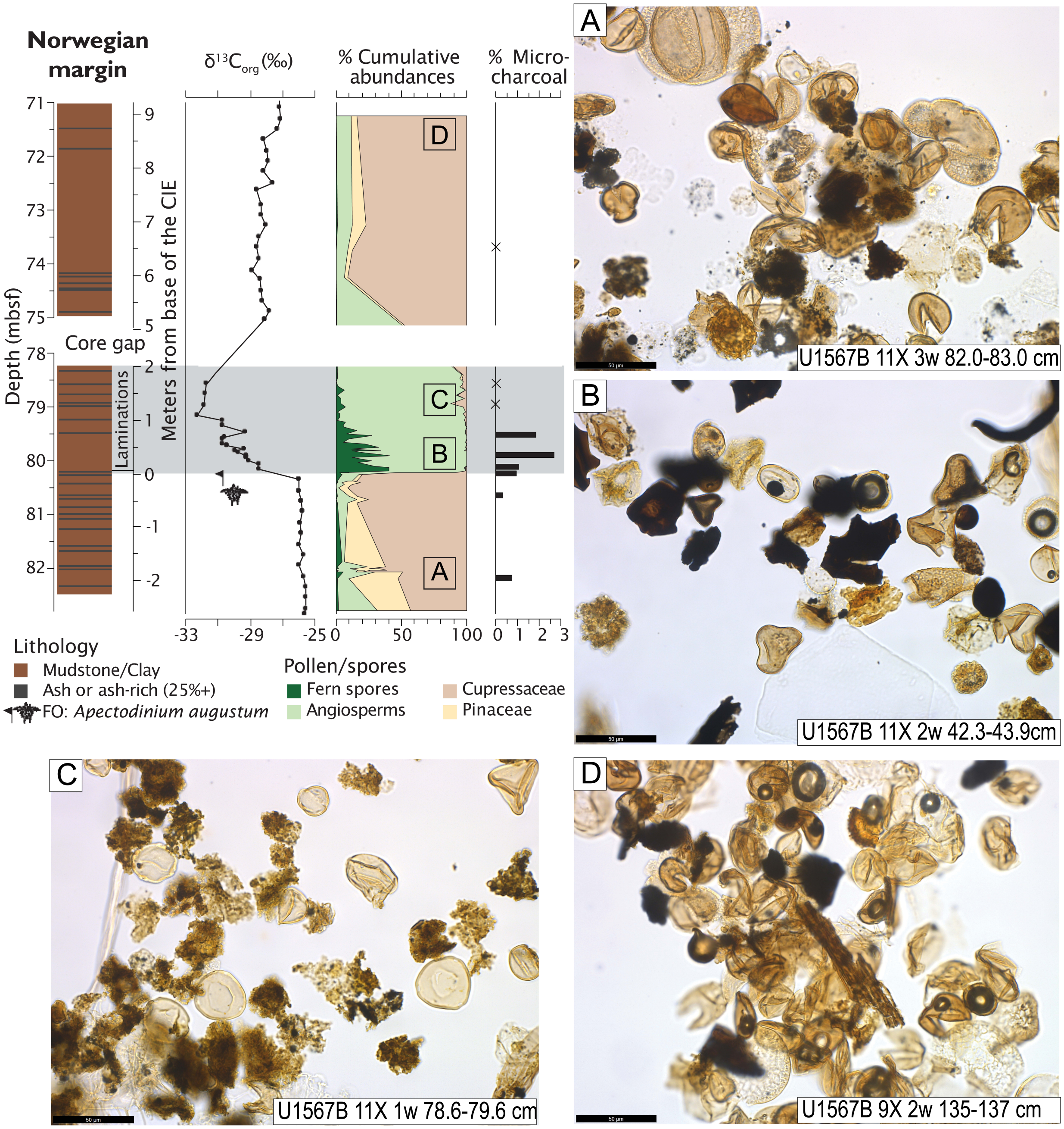

Fig. S3. Microscope images of palynomorphs from IODP X396, Norwegian Margin A) pre-CIE onset assemblages dominated by Cupressaceae (including *Taxodium* and *Metasequoia),* and the Pinaceae B) CIE onset fern-spike, phytoclasts and microcharcoal. C) Upper laminated interval, above CIE onset dominated by *Caryapollenites* and AOM-rich samples. D) CIE body assemblages dominated by Cupressaceae, mostly *Taxodium*.

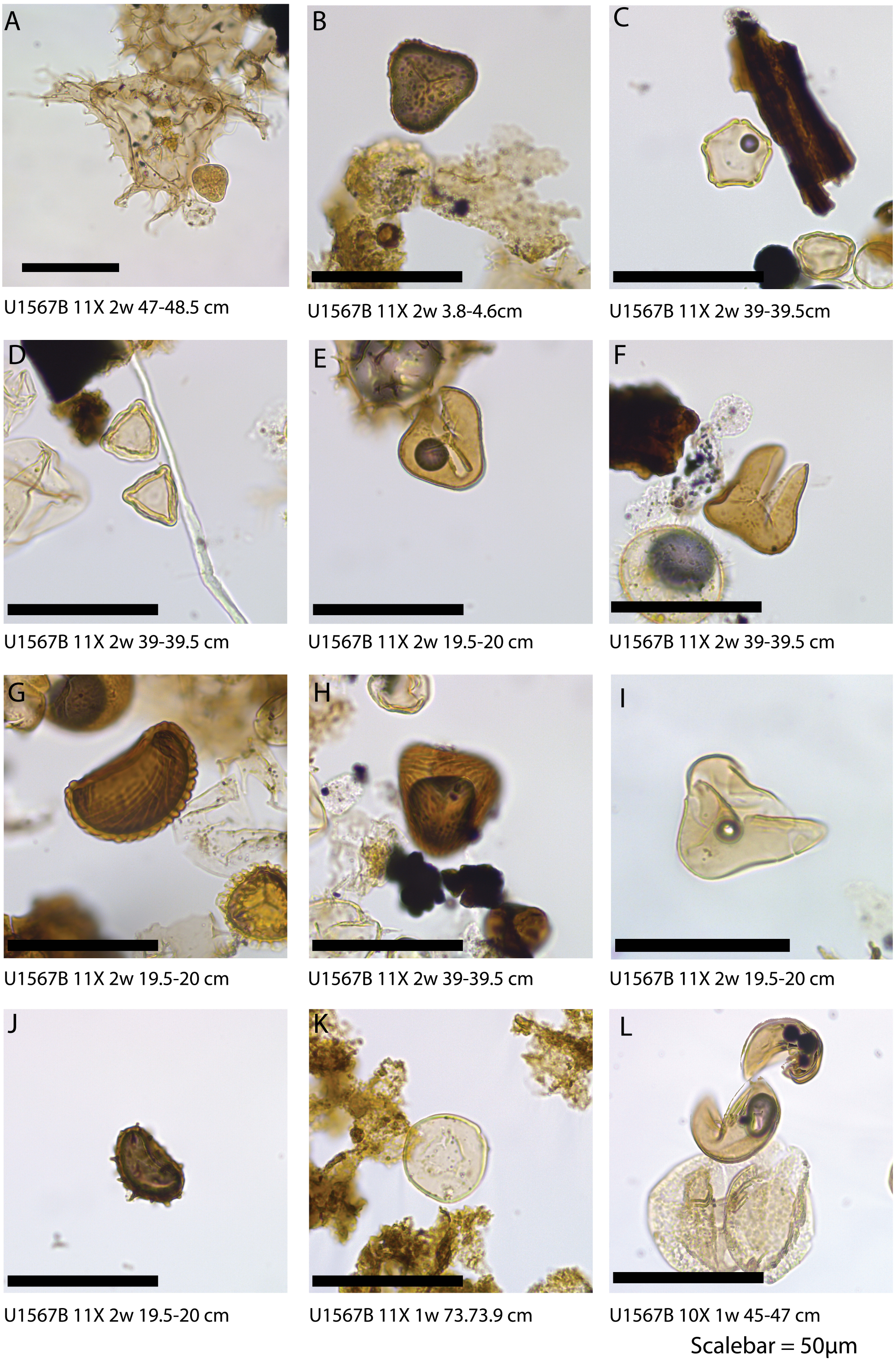

Fig. S4. Microscope images of palynomorphs from IODP X396, Norwegian Margin. A) the dinoflagellate cyst *Apectodinium augustum*, B) Pteridaceae, C) *Alnipollenites*, D) *Interpollis*, E & F) Gleicheniidites, G) Schizaeosporites, H) *Cicatricosisporites*, I) *Leiotriletes,* J) Polypodiaceae, K) *Caryapollenites*, L) Bisaccate and 2 Cupressaceae *(Taxodium)* pollen. Scale bar is 50µm in all images.

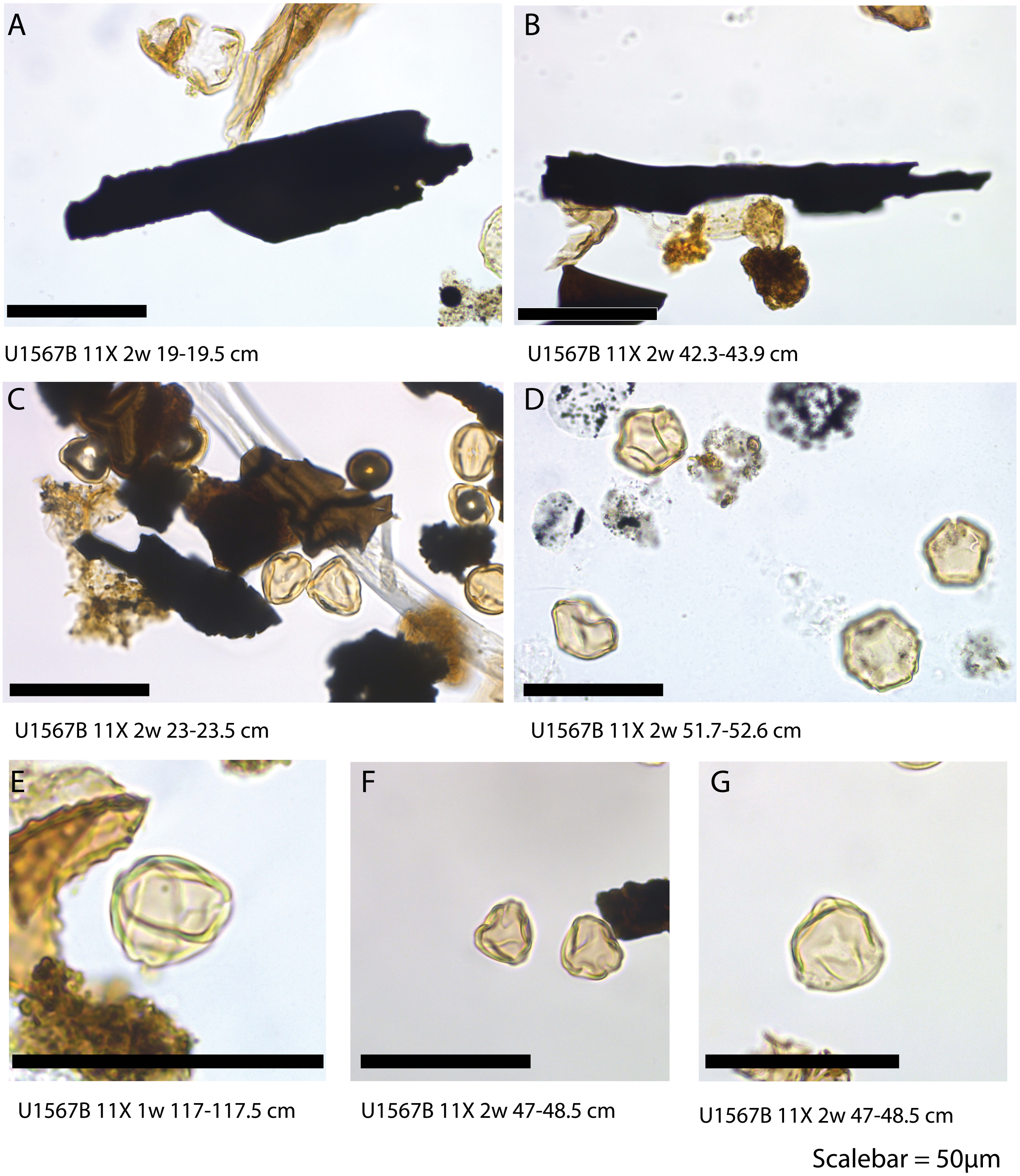

Fig. S5. Microscope images of palynomorphs from IODP X396, Norwegian Margin. A-C) microcharcoal, D) low diversity sample dominated by *Alnipollenites*. E) cf *Platycaryapollenites (P. triplicatus type?),* F) cf *P. swasticoides type?* G) *Platycaryapollenites anticyclus*

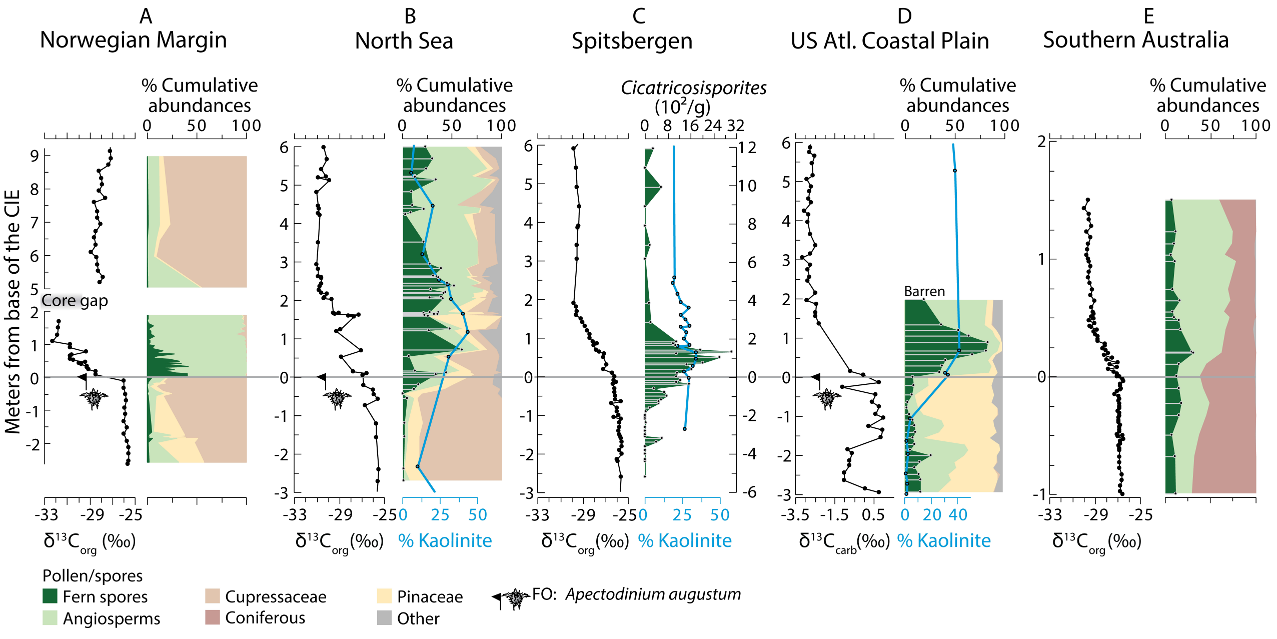

Fig. S6. CIE onset records aligned, showing the cumulative abundances of pollen and spores. Records aligned based on the FO of *A. augustum* and/or $\delta^{13}C$ values. A) At U1567B, Norwegian Marin, the base of the CIE is placed at 80.24 mbsf. B) In Hole 22/10a, North Sea the base of the CIE is placed at 2613.96m, supported by the FCO of *A.augustum* (updated from *(13)*. We excluded samples with *A.augustum* pre-CIE as this work used cuttings which may have contaminated downcore cuttings (see discussion in SI Text). C) On Spitsbergen, the base of the CIE was placed at 3.1m sampling height in the Longyearbyen section, of which bulk $\delta^{13}C_{org}$ values and number of *Cicatricosisporites* per gram dry weight are shown, depth axis shown on the left *(26–28)*. Spitsbergen kaolinite data is show from BH9/05, depth axis on the right *(27)*. D) At South Dover Bridge, New Jersey Shelf the base of the CIE was placed at 204.05m based on the FO of *A. augustum (32)*. E) At Point Margaret, Australia, the base of the CIE was placed at 50.8m *(36)*. Cumulative abundances of ferns spores, angiosperms and, Cupressaceae and Pinaceae shown as total abundance of pollen and spores assemblage. See paragraph '***Other sites and tie points'*** for full discussion of tie points.

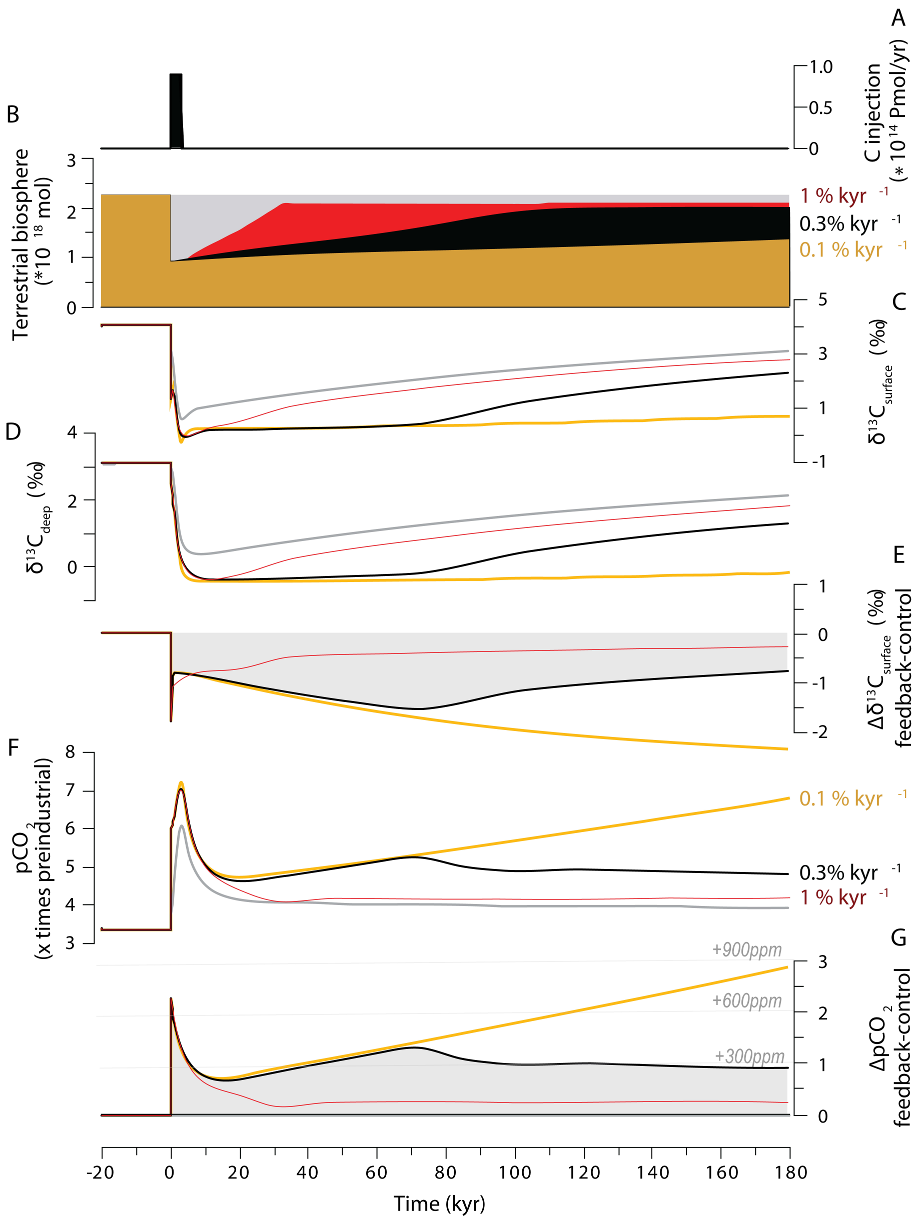

Fig. S7: Sensitivity tests of different terrestrial gross primary productivity (GPP) recovery rate scenarios. Model output data showing A) the endogenic C that is injected (0.8 Pg yr^{-1}). B) Three scenarios showing the terrestrial biosphere stock size when terrestrial GPP has a fast (1% per millennium, in red), intermediate (0.3% per millennium, in black) and a slower (0.1% per millennium, in yellow) recovery rate. In all scenarios, the maximum reduction of terrestrial GPP was 50%. The simulated carbon isotope excursions represent C) the global surface, D) deep-water masses, and E) the difference in $\delta^{13}C_{surf}$ between the control- and feedback scenarios. F) The modeled atmospheric CO_2 concentration plotted as x times pre-industrial and G) the difference in pCO_2 between the control- and feedback scenarios. The decline in atmospheric pCO_2 following the spike reflects carbon repartitioning among reservoirs, driven primarily by rapid ocean uptake. The control scenario is indicated with the grey line. In the main text, we also explore different scenarios for the maximum reduction in terrestrial GPP. Model details and further discussion provided in the Supplementary Method.

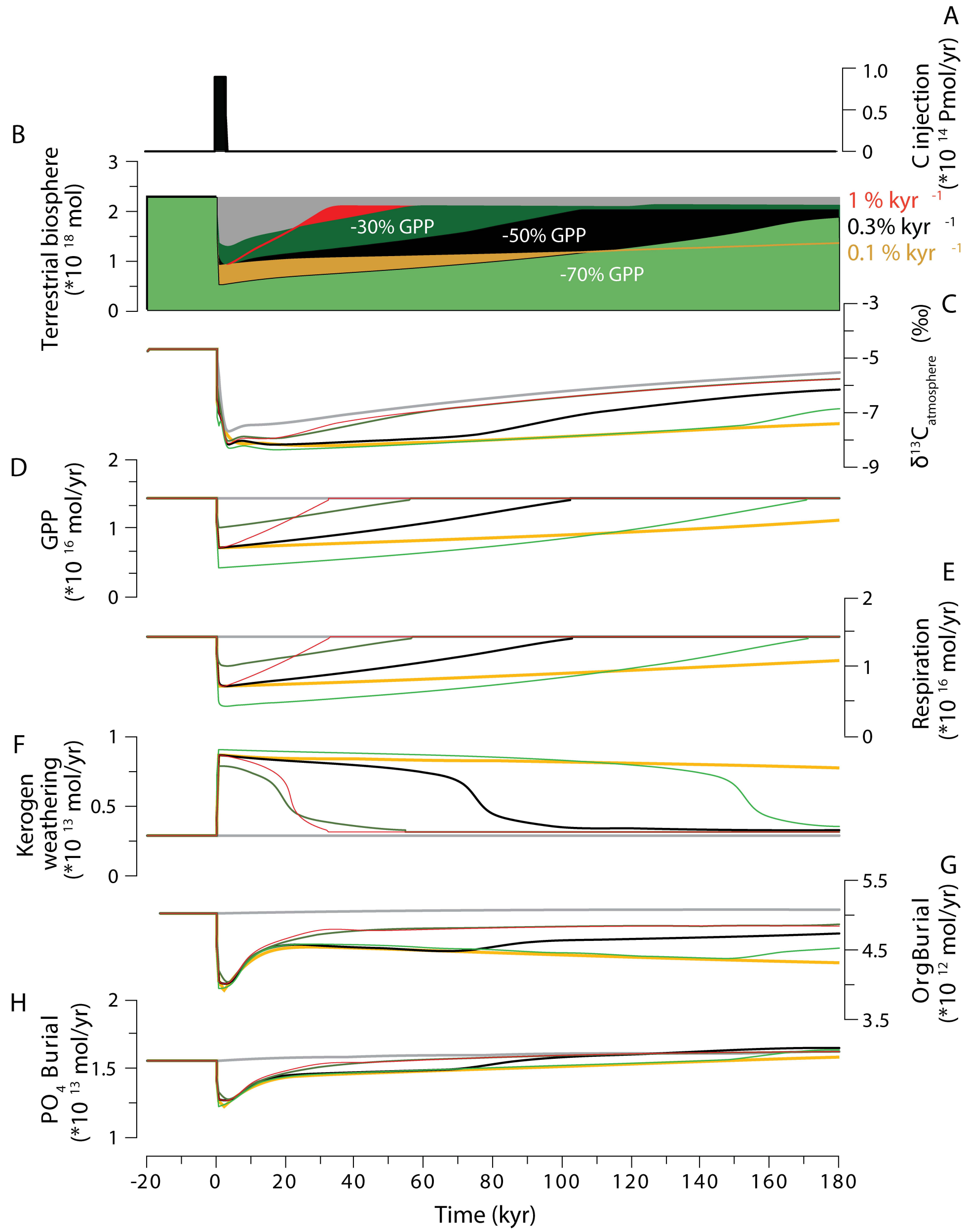

Fig. S8: Modelled changes in fluxes over time. Model output data showing A) the endogenic C that is injected (0.8 Pg yr^{-1}). The scenarios plotted in panels B-H include the control scenario in grey. We test various terrestrial feedback scenarios and the resulting size of the terrestrial biosphere stock size including a reduction in terrestrial GPP of 30% (in dark green, weakest response), 50% (in black, intermediate response) and 70% (in light green, strongest response) in response to endogenic C emissions. In these scenarios, the recovery rate of the terrestrial GPP was intermediate (0.3% per millennium, in black). We also test different recovery rates as indicated with a 1% recovery rate per millennium (faster recovery, in red) and 0.1% recovery rate per millennium (slow recovery in yellow). Plots show model output for each of these scenarios. B) the changes in terrestrial biosphere stock size, C) the modelled $\delta^{13}C_{atm,}$ D) changes in assimilation rate, reflecting global terrestrial GPP, E) respiration rate, F) kerogen weathering rate, G) Organic C burial and H) PO_4 burial. The units for changes in assimilation rate, respiration rate, kerogen weathering rate, Organic burial and PO_4 burial all in mol per year.

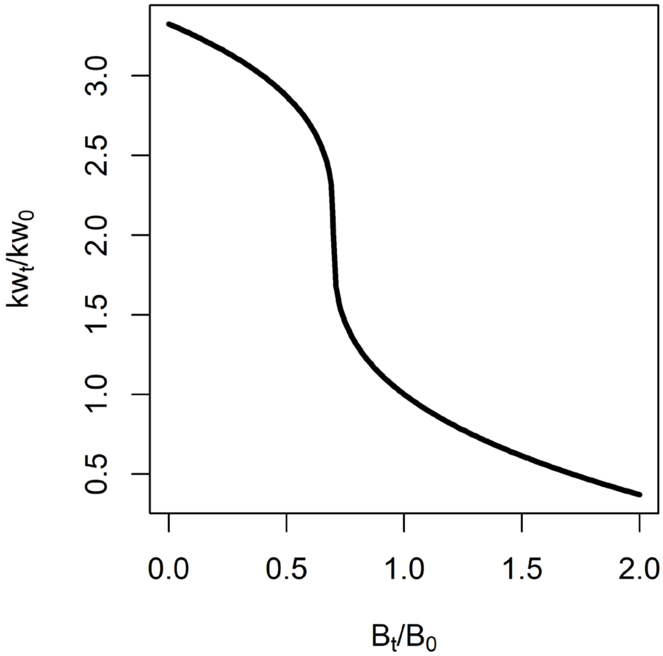

Fig. S9. Response of kerogen weathering flux (kw_t) to changes in the size of the terrestrial organic carbon stock (B_t). kw_t and kw_0 are the rate of the organic carbon flux from the lithosphere to the surface ocean (via rivers) at model timestep t. The weathering flux can flip between two semi-stables states, with a threshold value of B_t of 0.7, representing a threshold at 70% of the biospheric carbon stock at B_0. Below 70% of the biospheric carbon stock, kerogen weathering rates are hypothesized to be higher due to the decrease in landscape-stabilizing vegetation.

Dataset S1: Link to data:

https://zenodo.org/records/15230472?token=eyJhbGciOiJIUzUxMiJ9.eyJpZCI6IjczNzM4YjExLTQxNDktNGIwYS1iODM3LTM3MjA3OWY5NDBlNSIsImRhdGEiOnt9LCJyYW5kb20iOiIwMWRkMDI0YTdjMWJlOGJmMzFkYzFmZWUyZjBkMTYzOSJ9.pNT0a0dtTWnPK-wyH_oNKkqy4n-yZsKVslQf7UmQQOc9mzeU0HYoOWzYvRHjZsabirqGdhZ1OJB_3UI_ymDwDQ

Software S1: see reference (61)

SI References

1. A. K. Behrensmeyer, S. M. Kidwell, R. A. Gastaldo, Taphonomy and paleobiology. *Paleobiology* **26**, 103–147 (2000).

2. C. R. Hupp, Hydrology, geomorphology, and vegetation of Coastal Plain rivers in the southeastern United States. (2000).

3. D. Jolley, M. Vieira, S. Jin, D. B. Kemp, Palynofloras, palaeoenvironmental change and the inception of the Paleocene Eocene Thermal Maximum; the record of the Forties Fan, Sele Formation, North Sea Basin. (2022). https://doi.org/10.6084/m9.figshare.c.6080873.

4. D. W. Jolley, "Palynofloral evidence for the onset and cessation of eruption of the Faroe Islands lava field" in *Faroe Islands Exploration Conference: Proceedings of the 2nd Conference*, (Annales Societatis Scientiarum Færoensis, 2009), pp. 156–173.

5. S. L. Wing, E. D. Currano, Plant response to a global greenhouse event 56 million years ago. *American Journal of Botany* **100**, 1234–1254 (2013).

6. M. E. Collinson, *et al.*, Palynological evidence of vegetation dynamics in response to palaeoenvironmental change across the onset of the Paleocene-Eocene Thermal Maximum at Cobham, Southern England. *Grana* **48**, 38–66 (2009).

7. M. E. Collinson, J. J. Hooker, D. R. Groecke, "Cobham lignite bed and penecontemporaneous macrofloras of southern England: A record of vegetation and fire across the Paleocene-Eocene Thermal Maximum" in *Causes and Consequences of Globally Warm Climates in the Early Paleogene*, (Geological Society of America, 2003).

8. R. D. Pancost, *et al.*, Increased terrestrial methane cycling at the Palaeocene–Eocene thermal maximum. *Nature* **449**, 332–335 (2007).

9. C. Heilmann-Clausen, B. Schmitz, The late Paleocene thermal maximum $\delta^{13}C$ excursion in Denmark? *GFF* **122**, 70–70 (2000).

10. B. Schmitz, *et al.*, Basaltic explosive volcanism, but no comet impact, at the Paleocene–Eocene boundary: high-resolution chemical and isotopic records from Egypt, Spain and Denmark. *Earth and Planetary Science Letters* **225**, 1–17 (2004).

11. E. Steurbaut, *et al.*, "Palynology, paleoenvironments, and organic carbon isotope evolution in lagoonal Paleocene-Eocene boundary settings in North Belgium" in *Causes and Consequences of Globally Warm Climates in the Early Paleogene*, (Geological Society of America, 2003).

12. J. Bujak, D. Mudge, A high-resolution North Sea Eocene dinocyst zonation. *JGS* **151**, 449–462 (1994).

13. S. Kender, *et al.*, Marine and terrestrial environmental changes in NW Europe preceding carbon release at the Paleocene–Eocene transition. *Earth and Planetary Science Letters* **353–354**, 108–120 (2012).

14. A. J. Powell, H. Brinkhuis, J. P. Bujak, Upper Paleocene-Lower Eocene dinoflagellate cyst sequence biostratigraphy of southeast England. *Geological Society Special Publication* **101**, 145–183 (1996).

15. A. Sluijs, *et al.*, Environmental precursors to rapid light carbon injection at the Palaeocene/Eocene boundary. *Nature* **450**, 1218–1221 (2007).

16. J. S. Eldrett, D. R. Greenwood, M. Polling, H. Brinkhuis, A. Sluijs, A seasonality trigger for carbon injection at the Paleocene-Eocene Thermal Maximum. *Climate of the Past* **10**, 759–769 (2014).

17. J. Bujak, H. Brinkhuis, "Global warming and dinocyst changes across the Paleocene/Eocene Epoch boundary" in *Late Paleocene–Early Eocene Climatic and Biotic Events in the Marine and Terrestrial Records*, (Columbia University Press, 1998), pp. 277–295.

18. M. E. Collinson, D. C. Steart, A. C. Scott, I. J. Glasspool, J. J. Hooker, Episodic fire, runoff and deposition at the Palaeocene–Eocene boundary. *JGS* **164**, 87–97 (2007).

19. Y. Xie, F. Wu, X. Fang, Abrupt collapse of a swamp ecosystem in northeast China during the Paleocene–Eocene Thermal Maximum. *Palaeogeography, Palaeoclimatology, Palaeoecology* **595**, 110975 (2022).

20. X.-T. Wang, Z. Chen, L. Cui, X. Wang, Spatiotemporal evolution of wildfire activity during the Paleocene-Eocene Thermal Maximum in China. *Sci. China Earth Sci.* (2025). https://doi.org/10.1007/s11430-024-1472-5.

21. Z. Chen, Z. Ding, Z. Tang, X. Wang, S. Yang, Early Eocene carbon isotope excursions: Evidence from the terrestrial coal seam in the Fushun Basin, Northeast China. *Geophysical Research Letters* **41**, 3559–3564 (2014).

22. Y. Li, *et al.*, Eocene hyperthermal events in the terrestrial system: Geochronological and astrochronological constraints in the Fushun Basin, NE China. *Marine and Petroleum Geology* **139**, 105604 (2022).

23. J. Frieling, *et al.*, Identification of the Paleocene–Eocene boundary in coastal strata in the Otway Basin, Victoria, Australia. *Journal of Micropalaeontology* **37**, 317–339 (2018).

24. A. Sluijs, H. Brinkhuis, "A dynamic climate and ecosystem state during the Paleocene-Eocene Thermal Maximum: inferences from dinoflagellate cyst assemblages on the New Jersey Shelf" (2009).

25. S. Kirtland Turner, P. M. Hull, L. R. Kump, A. Ridgwell, A probabilistic assessment of the rapidity of PETM onset. *Nat Commun* **8**, 353 (2017).

26. I. C. Harding, *et al.*, Sea-level and salinity fluctuations during the Paleocene-Eocene thermal maximum in Arctic Spitsbergen. *Earth and Planetary Science Letters* **303**, 97–107 (2011).

27. H. Dypvik, *et al.*, The Paleocene–Eocene thermal maximum (PETM) in Svalbard — clay mineral and geochemical signals. *Palaeogeography, Palaeoclimatology, Palaeoecology* **302**, 156–169 (2011).

28. A. J. Charles, “Palaeoceanographic change during the Palaeocene/Eocene thermal maximum in Arctic Spitsbergen.” (2011).

29. A. Sluijs, G. R. Dickens, Assessing offsets between the δ13C of sedimentary components and the global exogenic carbon pool across early Paleogene carbon cycle perturbations. *Global Biogeochemical Cycles* **26** (2012).

30. A. J. Charles, *et al.*, Constraints on the numerical age of the Paleocene-Eocene boundary. *Geochemistry, Geophysics, Geosystems* **12** (2011).

31. Y. Cui, A. F. Diefendorf, L. R. Kump, S. Jiang, K. H. Freeman, Synchronous Marine and Terrestrial Carbon Cycle Perturbation in the High Arctic During the PETM. *Paleoceanography and Paleoclimatology* **36**, e2020PA003942 (2021).

32. M. Nelissen, A. Sluijs, D. A. Willard, H. Brinkhuis, A high-resolution late Paleocene–early Eocene organic-walled dinoflagellate cyst zonation of the United States Atlantic Coastal Plain. *Journal of Micropalaeontology* **44**, 431–467 (2025).

33. S. L. Lyons, *et al.*, Palaeocene–Eocene Thermal Maximum prolonged by fossil carbon oxidation. *Nature Geosci* **12**, 54–60 (2019).

34. L. G. Podrecca, M. Makarova, K. G. Miller, J. V. Browning, J. D. Wright, Clear as mud: Clinoform progradation and expanded records of the Paleocene-Eocene Thermal Maximum. *Geology* **49**, 1441–1445 (2021).

35. J. M. Self-Trail, *et al.*, Shallow marine response to global climate change during the Paleocene-Eocene Thermal Maximum, Salisbury Embayment, USA. *Paleoceanography* **32**, 710–728 (2017).

36. E. P. Huurdeman, *et al.*, Rapid expansion of meso-megathermal rain forests into the southern high latitudes at the onset of the Paleocene-Eocene Thermal Maximum. *Geology* **49**, 40–44 (2020).

37. C. Berndt, *et al.*, Shallow-water hydrothermal venting linked to the Palaeocene–Eocene Thermal Maximum. *Nat. Geosci.* **16**, 803–809 (2023).

38. E. W. Stokke, *et al.*, Rapid and sustained environmental responses to global warming: the Paleocene–Eocene Thermal Maximum in the eastern North Sea. *Climate of the Past* **17**, 1989–2013 (2021).

39. S. J. Kemp, M. A. Ellis, I. Mounteney, S. Kender, Palaeoclimatic implications of high-resolution clay mineral assemblages preceding and across the onset of the Palaeocene–Eocene Thermal Maximum, North Sea Basin. *Clay miner.* **51**, 793–813 (2016).

40. V. Pujalte, J. I. Baceta, B. Schmitz, A massive input of coarse-grained siliciclastics in the Pyrenean Basin during the PETM: the missing ingredient in a coeval abrupt change in hydrological regime. *Climate of the Past* **11**, 1653–1672 (2015).

41. D. A. Willard, H. Brinkhuis, NOAA/WDS Paleoclimatology - Palynological Data from Paleocene-Eocene Sediments from the Mid-Atlantic, USA. NOAA National Centers for Environmental Information. https://doi.org/10.25921/4G51-0912. Deposited 2025.

42. T. G. Gibson, L. M. Bybell, D. B. Mason, Stratigraphic and climatic implications of clay mineral changes around the Paleocene/Eocene boundary of the northeastern US margin. *Sedimentary Geology* **134**, 65–92 (2000).

43. V. A. Korasidis, S. L. Wing, Palynofloral Change Through the Paleocene-Eocene Thermal Maximum in the Bighorn Basin, Wyoming. *Paleoceanography and Paleoclimatology* **38**, e2023PA004741 (2023).

44. L. Handley, E. M. Crouch, R. D. Pancost, A New Zealand record of sea level rise and environmental change during the Paleocene–Eocene Thermal Maximum. *Palaeogeography, Palaeoclimatology, Palaeoecology* **305**, 185–200 (2011).

45. K. Kaiho, *et al.*, Latest Paleocene benthic foraminiferal extinction and environmental changes at Tawanui, New Zealand. *Paleoceanography* **11**, 447–465 (1996).

46. H. Khozyem, T. Adatte, J. E. Spangenberg, A. A. Tantawy, G. Keller, Palaeoenvironmental and climatic changes during the Palaeocene–Eocene Thermal Maximum (PETM) at the Wadi Nukhul Section, Sinai, Egypt. *JGS* **170**, 341–352 (2013).

47. L. Handley, *et al.*, Changes in the hydrological cycle in tropical East Africa during the Paleocene–Eocene Thermal Maximum. *Palaeogeography, Palaeoclimatology, Palaeoecology* **329–330**, 10–21 (2012).

48. D. C. Kelly, J. C. Zachos, T. J. Bralower, S. A. Schellenberg, Enhanced terrestrial weathering/runoff and surface ocean carbonate production during the recovery stages of the Paleocene-Eocene thermal maximum. *Paleoceanography* **20** (2005).

49. C. Robert, J. P. Kennett, Antarctic subtropical humid episode at the Paleocene-Eocene boundary: Clay-mineral evidence. *Geol* **22**, 211 (1994).

50. Z. Chen, Z. Ding, S. Yang, C. Zhang, X. Wang, Increased precipitation and weathering across the Paleocene-Eocene Thermal Maximum in central China. *Geochemistry, Geophysics, Geosystems* **17**, 2286–2297 (2016).

51. E. H. Hollingsworth, *et al.*, Spatial and Temporal Patterns in Petrogenic Organic Carbon Mobilization During the Paleocene-Eocene Thermal Maximum. *Paleoceanography and Paleoclimatology* **39**, e2023PA004773 (2024).

52. A. A. Baczynski, *et al.*, Distortion of carbon isotope excursion in bulk soil organic matter during the Paleocene-Eocene thermal maximum. *Geological Society of America Bulletin* **128**, 1352–1366 (2016).

53. J. C. G. Walker, J. F. Kasting, Effects of fuel and forest conservation on future levels of atmospheric carbon dioxide. (1992).

54. D. E. Archer, J. L. Morford, S. R. Emerson, A model of suboxic sedimentary diagenesis suitable for automatic tuning and gridded global domains. *Global Biogeochemical Cycles* **16**, 17-1-17–21 (2002).

55. G. J. Bowen, Up in smoke: A role for organic carbon feedbacks in Paleogene hyperthermals. *Global and Planetary Change* **109**, 18–29 (2013).

56. D. T. Harper, *et al.*, Long- and short-term coupling of sea surface temperature and atmospheric CO_2 during the late Paleocene and early Eocene. *Proc. Natl. Acad. Sci. U.S.A.* **121**, e2318779121 (2024).

57. T. Kaur, P. Sharathi Dutta, Critical rates of climate warming and abrupt collapse of ecosystems. *Proceedings of the Royal Society A* **478**, 20220086 (2022).

58. J. G. Canadell, R. B. Jackson, Eds., *Ecosystem Collapse and Climate Change* (Springer International Publishing, 2021).

59. E. H. Denis, *et al.*, Decreased soil carbon in a warming world: Degraded pyrogenic carbon during the Paleocene-Eocene Thermal Maximum, Bighorn Basin, Wyoming. *Earth and Planetary Science Letters* **566**, 116970 (2021).

60. J. M. Cotton, N. D. Sheldon, M. T. Hren, T. M. Gallagher, Positive feedback drives carbon release from soils to atmosphere during Paleocene/Eocene warming. *American Journal of Science* **315**, 337–361 (2015).

61. Gabriel J. Bowen, SPATIAL-Lab/paleoCC: Athyrium. (2025). https://doi.org/10.5281/ZENODO.16954107. Deposited 26 August 2025.
